# Tregs from human blood differentiate into nonlymphoid tissue–resident effector cells upon TNFR2 costimulation

**DOI:** 10.1172/jci.insight.172942

**Published:** 2024-01-30

**Authors:** Mark Mensink, Lotte J. Verleng, Ellen Schrama, George M.C. Janssen, Rayman T.N. Tjokrodirijo, Peter A. van Veelen, Qinyue Jiang, M. Fernanda Pascutti, Marie-Louise van der Hoorn, Michael Eikmans, Sander de Kivit, Jannie Borst

**Affiliations:** 1 Department of Immunology and Oncode Institute,; 2 Center for Proteomics and Metabolomics,; 3 Department of Immunology, and; 4 Department of Obstetrics and Gynaecology, Leiden University Medical Center, Leiden, Netherlands.

**Keywords:** Immunology, Bioinformatics, Costimulation, T cells

## Abstract

Tregs can facilitate transplant tolerance and attenuate autoimmune and inflammatory diseases. Therefore, it is clinically relevant to stimulate Treg expansion and function in vivo and to create therapeutic Treg products in vitro. We report that TNF receptor 2 (TNFR2) is a unique costimulus for naive, thymus-derived Tregs (tTregs) from human blood that promotes their differentiation into nonlymphoid tissue–resident (NLT-resident) effector Tregs, without Th-like polarization. In contrast, CD28 costimulation maintains a lymphoid tissue–resident (LT-resident) Treg phenotype. We base this conclusion on transcriptome and proteome analysis of TNFR2- and CD28-costimulated CD4^+^ tTregs and conventional T cells (Tconvs), followed by bioinformatic comparison with published transcriptomic Treg signatures from NLT and LT in health and disease, including autoimmunity and cancer. These analyses illuminate that TNFR2 costimulation promoted tTreg capacity for survival, migration, immunosuppression, and tissue regeneration. Functional studies confirmed improved migratory ability of TNFR2-costimulated tTregs. Flow cytometry validated the presence of the TNFR2-driven tTreg signature in effector/memory Tregs from the human placenta, as opposed to blood. Thus, TNFR2 can be exploited as a driver of NLT-resident tTreg differentiation for adoptive cell therapy or antibody-based immunomodulation in human disease.

## Abstract 2

TNFR2 is a driver of the key characteristics of effector/memory Treg cells in a wide range of non-lymphoid tissues in health and disease.

## Graphical abstract

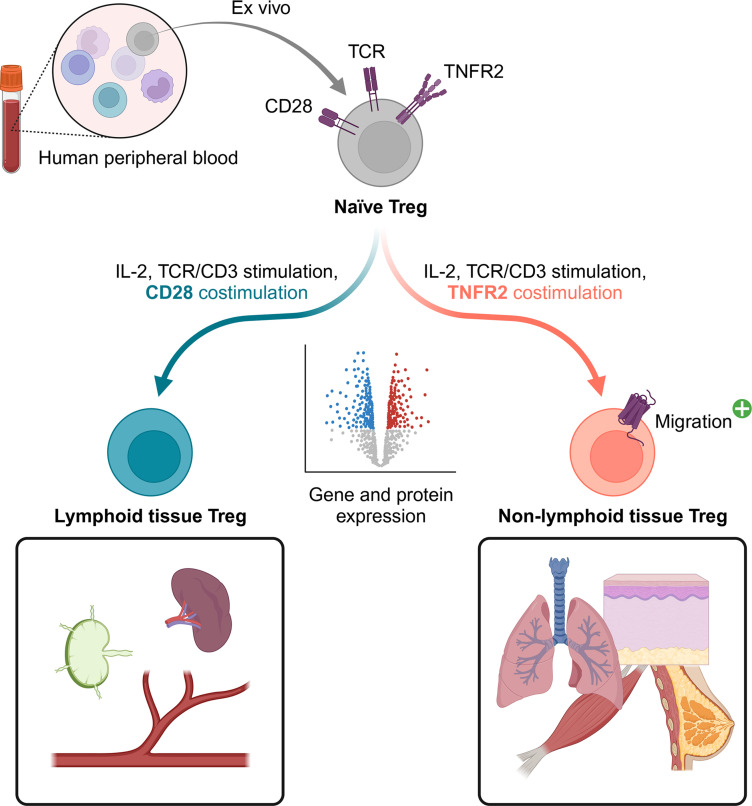

## Introduction

CD4^+^ Tregs are immunosuppressive and mediate immunological tolerance and tissue homeostasis. These core characteristics of Tregs are orchestrated by the transcriptional regulator FOXP3, in concert with other transcription factors and epigenetic modifiers (1), but Tregs exhibit diversity (2). Thymus-derived Tregs (tTregs) originate from immature, self-antigen–specific T cells in the thymus and circulate between blood and lymphoid tissues (LT). Upon activation, these cells can differentiate into effector Tregs and move to nonlymphoid tissues (NLT). Alternatively, activated CD4^+^ conventional T cells (Tconvs) can convert into peripherally induced Tregs (pTregs), primarily at mucosal surfaces and the maternal-fetal interface (3, 4). In both LT and NLT, Tregs protect against autoimmunity by suppressing responses of CD4^+^ and CD8^+^ Tconvs (5). In NLT, Tregs can adapt to local effector CD4^+^ Tconvs that produce lineage-specific cytokines characteristic of Th1, Th2, or Th17 cells. This process is associated with improved suppression of the corresponding Th cell responses (5, 6).

Tregs are attractive clinical targets to inhibit or induce Tconv responses, in order to attenuate or cure disease. Inhibiting Treg function can improve antitumor immunity (7), while promoting Treg function can ameliorate autoimmune or inflammatory diseases, transplant rejection, and graft-versus-host disease (GvHD). One key approach is to target specific cell surface receptors with antibodies, in order to deplete Tregs or modulate their activity (7). Another approach is to use adoptive Treg therapy to achieve immune suppression (8, 9). Such Treg therapies are in clinical trials for kidney transplantation (10), type 1 diabetes (11), inflammatory bowel disease (12), and GvHD (8). To make Treg products for adoptive transfer, Tregs are generally isolated from peripheral blood and expanded in vitro by T cell receptor (TCR)/CD3 stimulation and CD28 costimulation in presence of IL-2 (9). Key concerns in the field are how to instruct, in Tregs, functions such as long-term survival and migratory capacity; these functions are deemed required for treatment efficacy (9).

We here studied how either CD28- or TNFR2 (TNFRSF1B, CD120b) costimulation affects human tTregs, since accumulating evidence indicates that TNFR2 is important for Treg responses (13). The ligands of TNFR2 are transmembrane and soluble TNF, the former with higher affinity (13), and lymphotoxins (14). Compared with TNFR1, TNFR2 shows a much more restricted tissue distribution and does not induce cell death (15). TNFR2 is preferentially expressed on Tregs as opposed to Tconvs and facilitates Treg expansion and function (13, 16). Studies in mice proved that TNFR2 is important for Treg-mediated protection from autoimmune disease and GvHD (17–21). We recently discovered that TNFR2 specifically drives glycolysis in TCR/CD3-activated Tregs, providing the first evidence to our knowledge for TNFR2 as a metabolic regulator (22, 23).

We here present that CD3-mediated activation combined with TNFR2 costimulation endows peripheral blood–derived naive tTregs with key properties that characterize NLT-resident Tregs in human health and disease. The effects of TNFR2 costimulation stand in stark contrast with those of CD28 costimulation, which maintains an LT-resident Treg phenotype. Our conclusions are based on deep profiling of in vitro–stimulated, blood-derived tTregs by flow cytometry, transcriptomics, and proteomics and on comparing their gene expression programs with those of LT- and NLT-resident Tregs, as derived from a large range of studies in human health and disease. Our data identify TNFR2 as an important target in Treg-focused immunotherapies, since it instructs the Treg qualities desired for therapeutic efficacy.

## Results

### Human tTreg characteristics are conserved after TNFR2 or CD28 costimulation.

**Figure 1:**
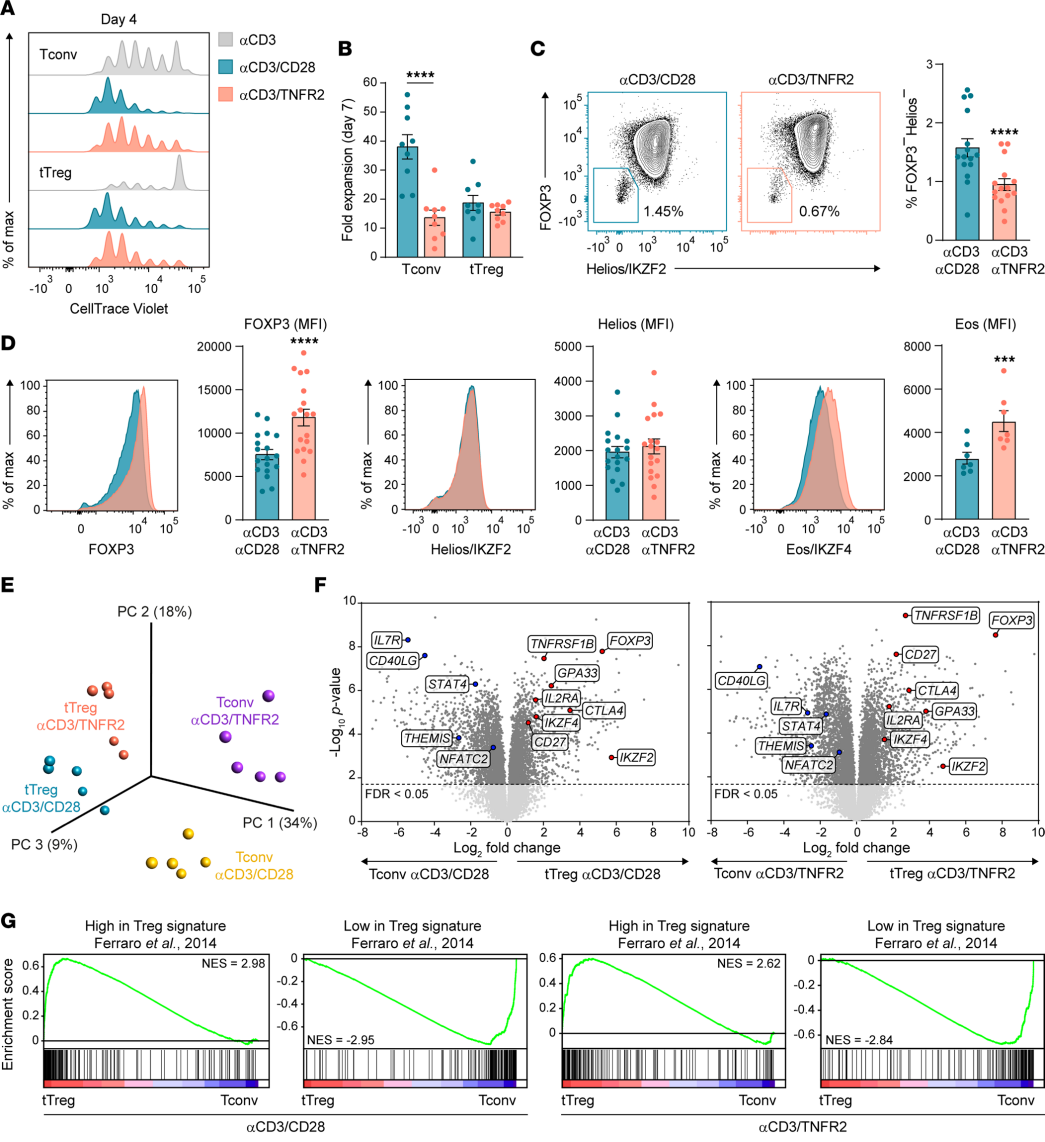
CD28 and TNFR2 costimulation differentially affect naive Tconvs and Tregs. Naive Tconvs and Tregs were purified from human peripheral blood and stimulated as indicated in Supplemental Figure 1. (**A**) Cell division assessed by CellTrace Violet (CTV) dilution in naive Tconvs and Tregs stimulated as indicated for 4 days (representative of *n* = 3). (**B**) Expansion of CD28- or TNFR2-costimulated Tconvs and Tregs, calculated by the ratio of live cell number on day 7 versus start of culture. Color legend as in **A**. Statistical analysis by 2-way ANOVA with Bonferroni’s post hoc test (*n* = 9). (**C**) Purity assessment of Treg cultures on day 7 by FOXP3 and Helios/IKZF2 expression, with representative plots (left) and FOXP3^–^Helios^–^ cell frequencies (right). Statistical analysis by paired 2-tailed Student’s *t* test (*n =* 15). (**D**) Flow cytometric analysis of FOXP3 (*n* = 18), Helios/IKZF2 (*n* = 18), and Eos/IKZF4 (*n* = 7) in tTregs on day 7. Representative plots and quantified protein expression by mean fluorescence intensity (MFI) are shown. Color legend as in **A**. Statistical analysis by paired 2-tailed Student’s *t* test. (**A**–**D**) Data are presented as mean ± SEM. Size (*n*) represents individual donors, analyzed in independent experiments. ***P < 0.001, ****P < 0.0001. (**E**) PCA of transcriptomics data from Tconvs and tTregs analyzed on day 7 (*n* = 5). (**F**) Volcano plots of transcriptomics data comparing CD28- or TNFR2-costimulated tTregs and Tconvs. Selected differentially expressed genes (FDR < 0.05) characteristic of Tconvs or Tregs are annotated. (**G**) GSEA evaluating the expression of signature genes of human Treg or Tconvs according to Ferraro et al. (27) in our transcriptomics data sets of CD28- or TNFR2-costimulated tTreg and Tconvs. Normalized enrichment scores (NES) are shown (FDR < 0.05).

For this study, naive CD4^+^ Tconvs and Tregs were sorted to high purity from peripheral blood of healthy donors based on a CD25^lo^CD127^hi^CD45RA^+^GPA33^int^ or a CD25^hi^CD127^lo^CD45RA^+^GPA33^hi^ phenotype, respectively (Supplemental Figure 1A; supplemental material available online with this article; https://doi.org/10.1172/jci.insight.172942DS1) (22, 24). To study the effect of TNFR2 versus CD28 costimulation on naive Tconvs and Tregs, sorted cells were cultured with agonistic mAb to CD3 alone or in combination with agonistic mAb to TNFR2 or CD28, in the presence of IL-2 (Supplemental Figure 1B). Naive Tregs required either TNFR2 or CD28 costimulation to proliferate well upon CD3-mediated activation, while Tconvs already proliferated upon CD3-mediated activation alone (Figure 1A). Based on live cell counts, Tregs expanded equally well upon either type of costimulation, while Tconvs expanded much better upon CD28 costimulation (Figure 1B). CD3-activated tTregs responded similarly to soluble TNF as to agonistic mAb to TNFR2, while both types of costimuli had significantly less effect on CD3-activated Tconvs (Supplemental Figure 1C). These data are in agreement with earlier findings that TNFR2 costimulation preferentially expands Tregs compared with Tconvs (16).

To assess nature and purity of the Treg culture, we examined expression of 3 transcription factors: the general Treg marker FOXP3 (1), the tTreg marker Helios (IKZF2), and the supporter of Treg stability Eos (IKZF4) (25, 26). On day 7, the Treg expansion culture consisted of over 98% of FOXP3^+^Helios^+^ tTregs, with the remainder being FOXP3^–^Helios^–^ Tconvs (Figure 1C). Furthermore, FOXP3 and Eos expression were higher in tTregs after TNFR2 as compared with CD28 costimulation, while Helios expression did not differ (Figure 1D). TNFR2- and CD28-costimulated tTregs suppressed anti-CD3–driven Tconv proliferation in equal measure (Supplemental Figure 1D).

To comprehensively examine the effect of TNFR2 versus CD28 costimulation on tTregs and Tconvs, we performed bulk transcriptome analysis on day 7. Tconvs and tTregs were evidently distinct from one another in terms of gene expression and differentially responded to CD28 or TNFR2 costimulation, as revealed by principal component analysis (PCA) (Figure 1E). tTregs and Tconvs showed large differences in cell type–specific gene expression — such as higher levels of transcription factors *FOXP3*, *IKZF2* (Helios) and *IKZF4* (Eos) in tTregs — after either TNFR2 or CD28 costimulation (Figure 1F). Other genes characteristic of Tregs were also differentially expressed, including *CTLA4*, *IL2RA* (CD25), and *GPA33* that we based our cell sorting on (22, 24). Compared with Tconvs, tTregs also expressed higher levels of transcripts for *TNFRSF1B* (TNFR2) and *CD27* (TNFRSF7). In contrast, *IL7R* (CD127), *CD40LG*, *THEMIS*, and *STAT4* were highly expressed in Tconvs, in line with literature (27).

To validate Treg and Tconv identity in an unbiased manner, we performed gene set enrichment analysis (GSEA), using as a reference a robust data set of differentially expressed transcripts in human Tregs versus Tconvs, reported by Ferraro et al. (27) (Figure 1G). In the GSEA depicted, black bars represent Treg signature genes from this data set, positioned by their rank in our comparative transcriptomes. The green curve indicates the enrichment score. Genes with high expression in the Treg signature of Ferraro et al. were enriched in tTregs, while genes with low expression in this Treg signature were enriched in Tconvs, regardless of CD28 or TNFR2 costimulation (Figure 1G) (27). The collective data show that, upon TNFR2 or CD28 costimulation, human tTregs expand and acquire differential gene expression but maintain their unique identity compared with Tconvs.

### TNFR2 costimulation increases cell surface expression of functionally relevant proteins on tTregs.

**Figure 2:**
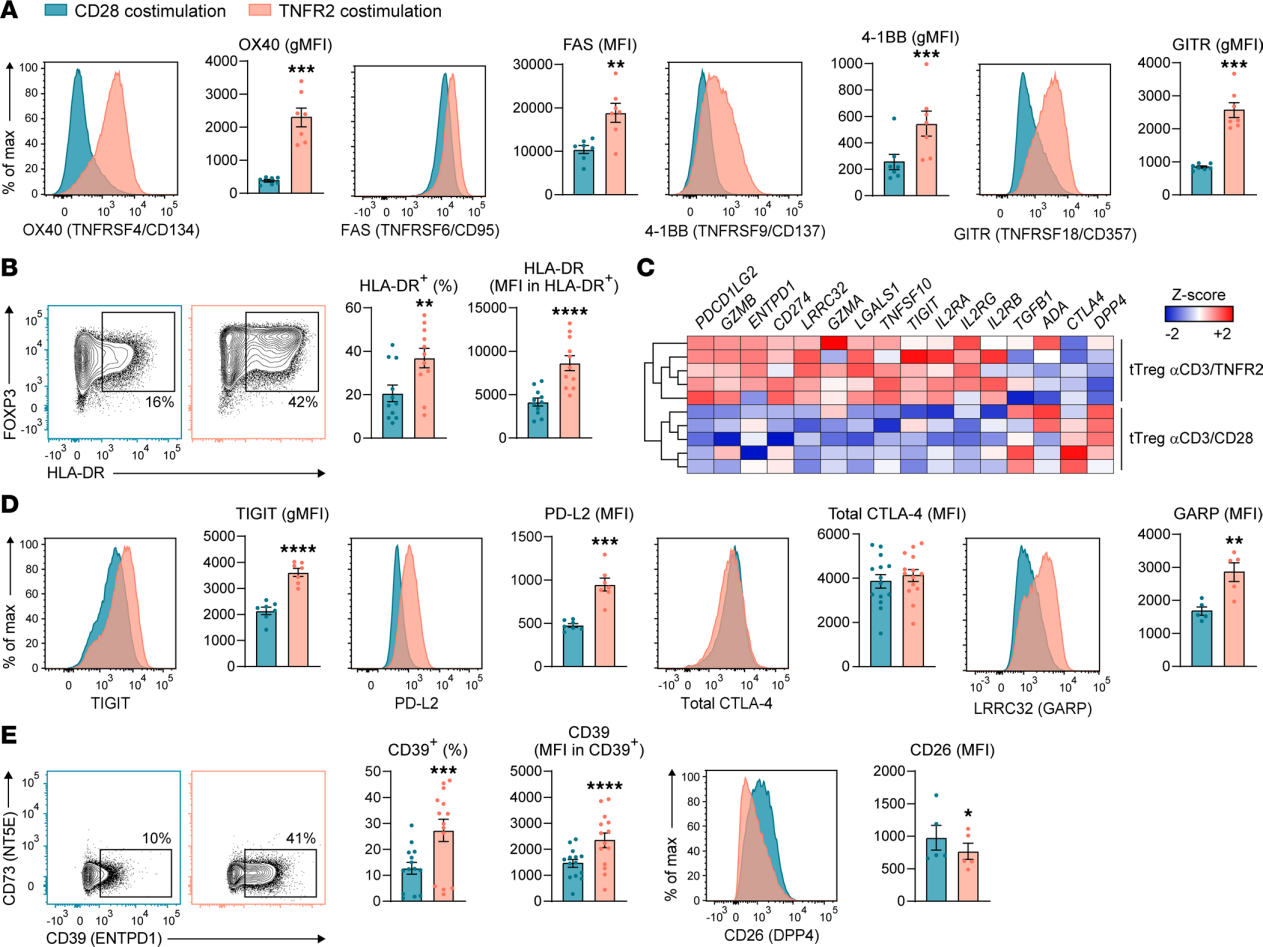
TNFR2 costimulation specifically upregulates cell surface expression of proteins involved in cell survival and suppressive functions on tTregs. (**A** and **B**) Naive tTregs were activated with anti-CD3 mAb and costimulated with agonistic mAb to either CD28 or TNFR2 for 7 days and analyzed by flow cytometry. Representative plots and quantified protein expression are shown for OX40, FAS, 4-1BB, GITR (all *n* = 7) (**A**), and HLA-DR (*n* = 11) (**B**). Data are quantified as MFI, geometric MFI (gMFI), or percentage of positive expression as indicated by gates. MFI within the positive fraction is shown for HLA-DR. (**C**) Heatmap based on the transcriptomics data outlined in Figure 1 showing selected genes involved in (potential) suppressive mechanisms described for Tregs. *Z* scores are color coded. (**D**) Cell surface expression of TIGIT (*n* = 7), PD-L2 (*n* = 7), and LRRC32 (GARP) (*n* = 5) and total expression of CTLA-4 in permeabilized cells (*n* = 14) as determined by flow cytometry after stimulation of tTregs as indicated for **A** and **B**. (**E**) Cell surface expression of CD39 (ENTPD1), CD73 (NT5E), and CD26 (DPP4) analyzed after stimulation of tTregs as indicated for **A** and **B**. CD39 data are quantified by depicting the percentage of positive expression as indicated by gates, as well as the MFI within the positive fraction (*n* = 14). CD26 data are quantified as MFI (*n* = 5). (**A**, **B**, **D**, and **E**) Statistical analysis was done by paired 2-tailed Student’s *t* test. Data are presented as mean ± SEM. Sample size (*n*) represents individual donors, analyzed in independent experiments. *P < 0.05, **P < 0.01, ***P < 0.001, ****P < 0.0001.

We used flow cytometry to further determine the effect of CD28 versus TNFR2 costimulation on expression of proteins that have been implicated in Treg phenotype and function. Compared with CD28 costimulation, TNFR2 costimulation with agonistic mAb upregulated cell surface expression of the TNFRSF members OX40 (TNFRSF4/CD134), FAS (TNFRSF6/CD95), 4-1BB (TNFRSF9/CD137), and GITR (TNFRSF18/CD357) (Figure 2A and Supplemental Figure 2). These receptors were likewise upregulated by soluble TNF in a dose-dependent manner (Supplemental Figure 3). All TNFRSF members associate with intracellular TRAF signaling adaptors and can thereby activate NF-κB signaling and promote cell survival. TNFRSF members are important costimulatory receptors for T cells that mediate cell survival in LT and NLT and can also regulate cell migration and differentiation (28). Among these TNFRSF members, FAS is the only death receptor that can mediate both pro- and antiapoptotic signaling (29). TNFR2 costimulation also increased HLA-DR expression on tTregs (Figure 2B), as suggested by our transcriptomics data (Supplemental Table 1) and in line with earlier findings (30). HLA-DR reportedly marks Tregs with high suppressive potential (31).

Upon selective examination of genes involved in suppressive functions of Tregs, it was apparent from the transcriptomics data that TNFR2 compared with CD28 costimulation increased mRNA levels of a variety of such genes in tTregs (Figure 2C). Follow-up by flow cytometry showed that TNFR2 costimulation increased cell surface expression of the coinhibitory receptor TIGIT that can enhance the suppressive function of Tregs (32), as well as PD-L2, a ligand for the coinhibitory receptor PD-1 (Figure 2D). We did not observe altered expression of total (cell surface and intracellular) CTLA-4 (Figure 2D). At the transcript level, expression of the suppressive molecule *TGFB1* was not specifically increased upon TNFR2 costimulation, but expression of *LRRC32* (GARP), that activates latent TGF-β (33), was specifically increased (Figure 2C). Flow cytometry confirmed increased cell surface expression of LRRC32 protein on tTregs after TNFR2 costimulation (Figure 2D), suggesting an increased ability of tTregs to suppress via TGF-β. Tregs can also suppress immune responses by CD39-mediated (ENTPD1-mediated) hydrolysis of proinflammatory ATP into ADP and AMP. This hydrolysis step can be followed by CD73-mediated (NT5E-mediated) hydrolysis of AMP into immunosuppressive adenosine (34, 35). CD39/ENTPD1 expression was specifically upregulated in TNFR2-costimulated tTregs at the mRNA level (Figure 2C) and cell surface–protein level (Figure 2E and Supplemental Figure 3). CD73/NT5E was not detected at the mRNA or protein level in cultured tTregs (Figure 2E). The cell surface enzymes ADA and CD26 (DPP4) interact with each other to convert immunosuppressive adenosine into inosine. Low transcript levels and low CD26/DPP4 protein levels argue that TNFR2-costimulated tTregs do not favor this conversion (Figure 2, C and E). The observed tTreg phenotype ties in with reported data that Tregs cooperate with other CD73^+^ cells, such as Tconvs or B cells, to generate adenosine by each performing a step in the hydrolysis pathway via CD39 and CD73, respectively (34, 35).

### TNFR2 costimulation increases tTreg survival/proliferation and migration/adhesion according to transcriptome and proteome analysis.

**Figure 3:**
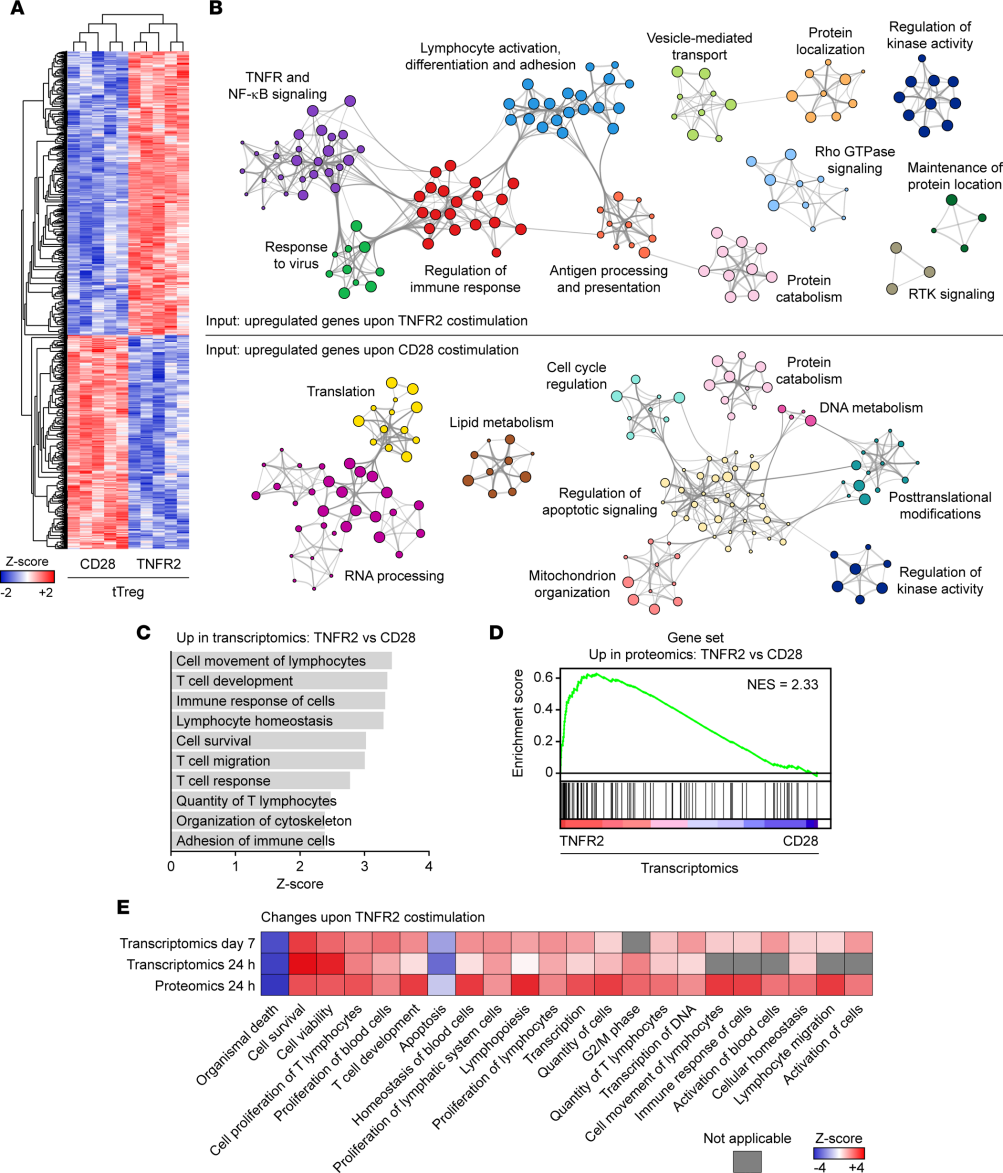
CD28 and TNFR2 costimulation differentially affect biological processes in tTregs. (**A**) Heatmap showing 1,229 genes differentially expressed between TNFR2- and CD28-costimulated tTregs, based on transcriptome analysis outlined in Figure 1 (FDR < 0.05). (**B**) Overrepresentation network classifying differentially expressed genes into functional categories (enrichment factor > 1.5, *P* < 0.01). Nodes represent biological process terms from GO or Reactome. Highly connected terms were grouped, colored, and annotated manually by a shared term. (**C**) Upregulated cellular functions in TNFR2- versus CD28-costimulated tTregs according to IPA of the same transcriptomics data (FDR < 0.05). (**D**) GSEA using proteins upregulated according to proteome analysis in preexpanded tTregs after 24 hours of restimulation by anti-CD3 mAb and TNFR2 versus CD28 costimulation (*P* < 0.05, 1.25-fold change). Enrichment is shown in the transcriptome of tTregs costimulated via TNFR2 for 7 days (FDR < 0.05). (**E**) Heatmap showing similar changes in cellular processes across multiple omics analyses according to IPA. Comparative analysis includes transcriptomics data outlined in **A** (Transcriptomics day 7, αCD3/TNFR2 versus αCD3/CD28; FDR < 0.05), published transcriptomics data from preexpanded tTregs restimulated for 24 hours (Transcriptomics 24 hours, αCD3/TNFR2 versus αCD3; FDR < 0.05) (22, 23), and proteomic data from restimulated tTregs (Proteomics 24 hours, αCD3/TNFR2 versus αCD3/CD28; *P* < 0.05, 1.1-fold change). *Z* scores are color coded.

Next, we compared the differential effect of TNFR2 versus CD28 costimulation on tTreg functionality, as apparent from the transcriptome analysis. Between these 2 groups, 1,229 genes were differentially expressed, consisting of 701 upregulated genes and 528 downregulated genes specific for TNFR2 costimulation (Figure 3A and Supplemental Table 1). Analysis of associated biological processes by Gene Ontology (GO)/Reactome analysis indicated that TNFR2 costimulation promoted in tTregs TNFR/NF-κB signaling, activation, differentiation and adhesion, immune response regulation, and various adaptations in signal transduction- and cell biological features (Figure 3B, top). After CD28 costimulation, tTregs responded primarily by activating biosynthetic processes, including ribosome and mitochondrial biogenesis and protein translation, next to metabolism and cell cycle (Figure 3B, bottom). GSEA using the Hallmark gene set collection also identified TNF signaling via NF-κB specifically in the transcriptome of TNFR2-costimulated tTregs, as well as IFN and cytokine signaling, while CD28 costimulation induced MYC targets, in line with the promotion of biosynthetic and metabolic processes required for cellular activation and proliferation (Supplemental Figure 4, A and B) (36). Ingenuity Pathway Analysis (IPA) of cellular functions also identified a specific effect of TNFR2 costimulation on tTreg survival, migration, adhesion, cytoskeletal changes, and responsiveness (Figure 3C). Molecules associated with lymphocyte survival regulated by TNFR2 included *TNFRSF4* (OX40), *TNFRSF9* (4-1BB), *TRAF2* and *-3*, *TNFAIP3*, and various NF-κB pathway components such as *TANK*, *MAP3K14*/*NIK*, *NFKB1*, *NFKB2*, and *RELB* (Supplemental Figure 4C).

To examine tTreg responses at the protein level, we performed proteomics on preexpanded tTregs that were stimulated via CD3 and costimulated via TNFR2 or CD28 for 24 hours. This analysis identified 94 upregulated proteins and 28 downregulated proteins upon TNFR2 costimulation (Supplemental Figure 4D). These proteins included TNFRSF4/OX40, TNFRSF18/GITR, FOXP3, ICAM-1, and various TNFR-associated signaling components. GSEA showed concordance between the effects of TNFR2 and CD28 costimulation at the protein- and mRNA level, even though the proteomic data were from preexpanded tTregs at 24 hours and the transcriptomics data were from freshly isolated cells on day 7 (Figure 3D). In agreement, TNFR2 costimulation led to similar transcriptomic changes in preexpanded or freshly isolated tTregs at 24 hours or 7 days, respectively (Supplemental Figure 4E) (22, 23). Importantly, IPA of changes in mRNA and protein expression both indicated that TNFR2 as compared with CD28 costimulation specifically increased tTreg activation, survival, proliferation, and migration (Figure 3E).

### tTregs show enhanced migration upon TNFR2 costimulation.

**Figure 4:**
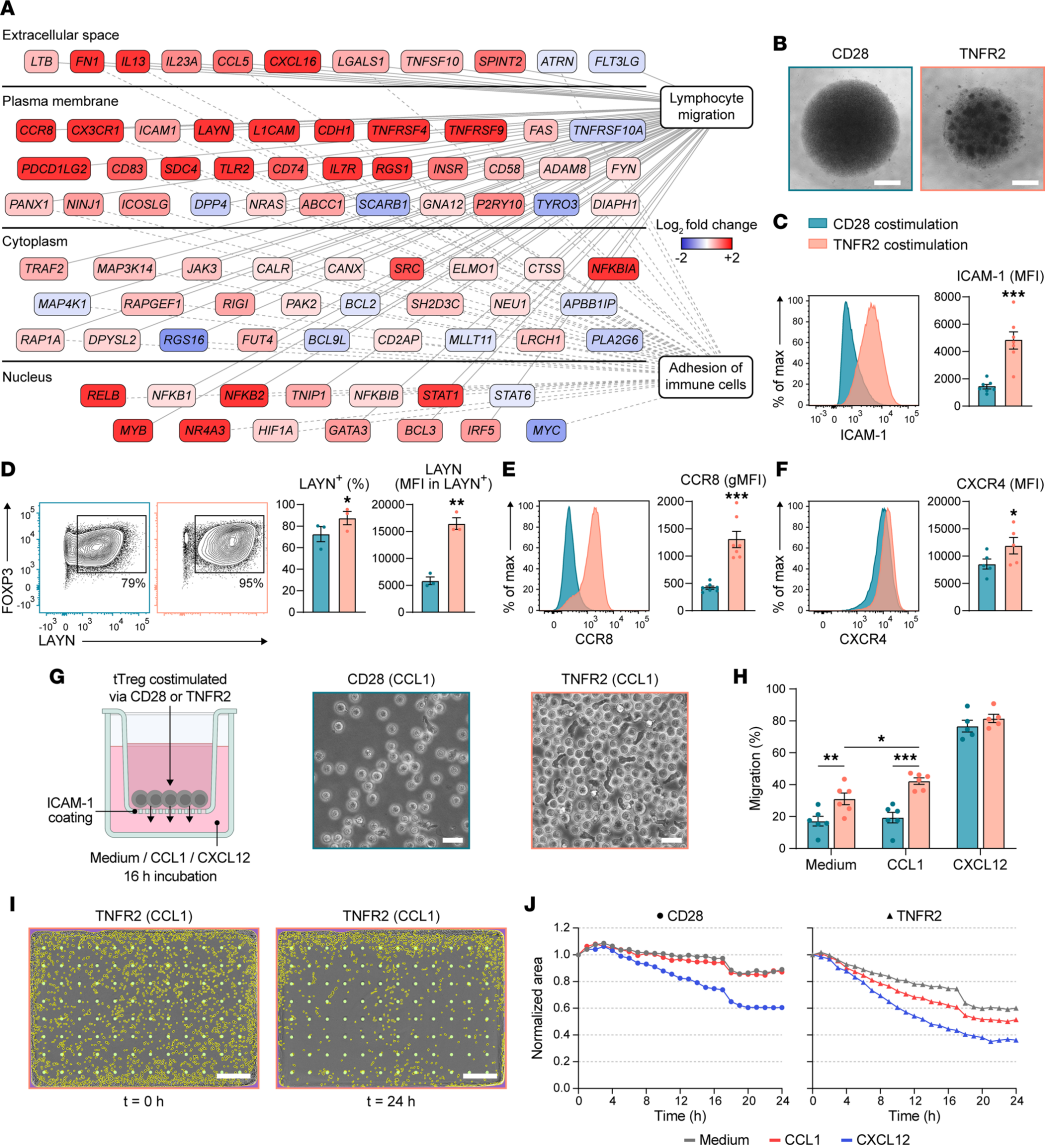
TNFR2 costimulation upregulates the migratory capacity of tTregs. (**A**) Differentially expressed genes associated with lymphocyte migration and/or adhesion, identified by IPA as upregulated in TNFR2- versus CD28-costimulated tTregs. Log_2_ fold changes are color coded. (**B**) Light microscopy images of differentially costimulated tTreg cultures on day 7. Scale bar: 500 μm. (**C**–**F**) Flow cytometric analysis of differentially costimulated tTregs on day 7 of culture. Representative plots and quantification are shown for ICAM-1 (*n* = 7) (**C**), LAYN (*n* = 3) (**D**), CCR8 (*n* = 7) (**E**), and CXCR4 (*n* = 5) (**F**). Data are quantified as MFI, gMFI, or percentage positive. Statistical analysis by paired 2-tailed Student’s *t* test. (**G**) Left: Schematic depiction of migration assay using Transwell inserts with 5 μm pore size to assess tTreg migration on day 7. Right: Light microscopy images at 16 hours, showing differentially costimulated tTregs in the bottom chamber containing CCL1. Scale bar: 20 μm. (**H**) Frequency of migrated cells after the 16-hour assay described in **G**. Statistical analysis by 2-way repeated-measures ANOVA with Tukey’s post hoc test was done on indicated groups (*n* = 6), without incorporating CXCL12 data (*n* = 5). (**C**–**F** and **H**) Data are presented as mean ± SEM. Group size (*n*) represents individual donors, analyzed in independent experiments. *P < 0.05, **P < 0.01, ***P < 0.001. (**I**) Migration assay using an Incucyte Clearview 96-well plate, showing images of TNFR2-costimulated tTregs cultured in the presence of CCL1 at 0 and 24 hours. Detected cells and pores are indicated in yellow and light green, respectively. Scale bar: 400 μm. (**J**) Migration as quantified by the area occupied by tTregs on the insert, normalized to 0 h (representative of *n* = 2).

Transcriptome analysis indicated that TNFR2 costimulation specifically upregulated migration and adhesion in tTregs (Figure 4A). Differentially expressed genes associated with these processes included chemokine receptors *CCR8* and *CX3CR1* and adhesion molecules fibronectin (*FN1*), *ICAM1*, *L1CAM*, *CDH1*, and *LAYN* (Figure 4A). Morphological changes as observed by light microscopy confirmed increased adhesion of TNFR2-costimulated tTregs (Figure 4B). Analysis by flow cytometry validated that, compared with CD28 costimulation, TNFR2 costimulation upregulated cell surface expression of ICAM-1 (Figure 4C) that, upon binding to integrin LFA-1 (αLβ2), mediates T cell adhesion (37). Furthermore, TNFR2 costimulation of tTregs upregulated LAYN (Figure 4D), a receptor that binds hyaluronic acid and promotes focal adhesions (38). LAYN is reportedly highly expressed on tumor-infiltrating Tregs and mediates adhesion of Tregs in the skin (38, 39). TNFR2 costimulation of tTregs also upregulated cell surface expression of CCR8 (Figure 4E and Supplemental Figure 2), a receptor for the chemokines CCL1 and CCL18 that is highly expressed on Tregs in healthy NLT and tumors (40, 41). Like TNFR2 agonist, soluble TNF increased ICAM-1 and CCR8 cell surface expression on tTregs (Supplemental Figure 3).

Next, we directly tested the effect of TNFR2 versus CD28 costimulation on tTreg migration. Migration to CXCL12 was included as a positive control, since both CD28- and TNFR2-costimulated tTregs expressed its receptor CXCR4 (Figure 4F). Transwell inserts were coated with ICAM-1 to facilitate cell adhesion, and CD28- or TNFR2-costimulated tTregs were tested for chemotactic migration toward low-serum culture medium with or without CCL1 (CCR8 ligand) or CXCL12 (CXCR4 ligand) (Figure 4G). Morphological changes toward a more elongated cell shape were particularly observed among transmigrated tTregs that had received TNFR2 costimulation (Figure 4G). Compared with CD28 costimulation, TNFR2 costimulation significantly increased tTreg migration toward low-serum culture medium, suggesting increased basal motility (Figure 4H). Moreover, TNFR2 costimulation significantly increased tTreg migration toward CCL1, while tTregs showed similar migration toward CXCL12 upon either TNFR2 or CD28 costimulation (Figure 4H). These findings were corroborated by an imaging-based kinetic analysis of tTreg migration, showing that TNFR2 costimulation increases the migratory capacity of tTregs as compared with CD28 costimulation (Figure 4, I and J).

### Costimulated naive tTregs become effector tTregs without Th-type differentiation.

**Figure 5:**
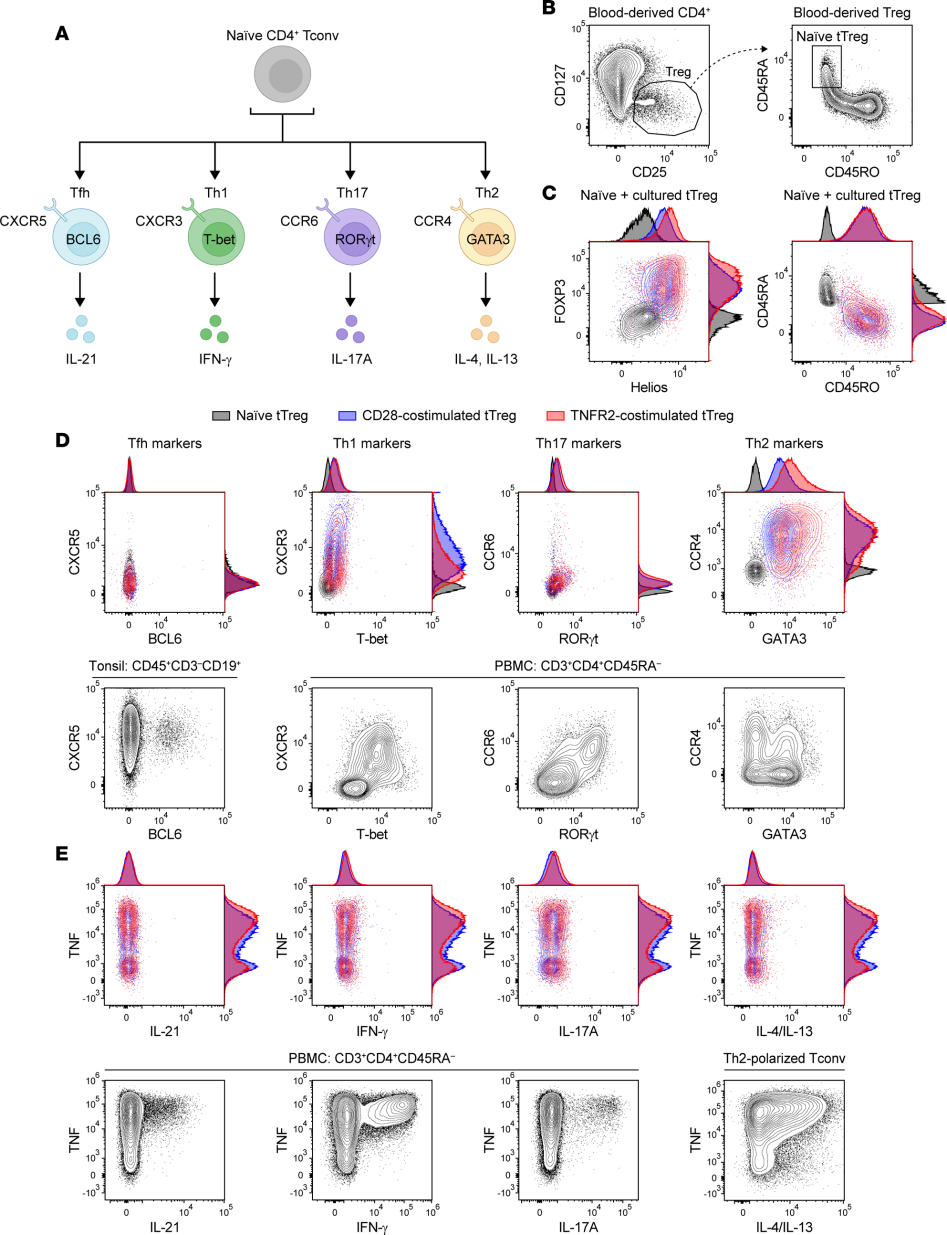
Costimulated tTregs acquire an effector Treg profile without Th-like polarization. (**A**) Scheme depicting naive CD4^+^ Tconv effector differentiation into Tfh, Th1, Th17, and Th2 lineages, with defining transciption factors, chemokine receptors, and cytokines. (**B**) Representative plots showing gating strategy to identify CD25^hi^CD127^lo^ Tregs among CD4^+^ T cells from PBMCs (left) and gating strategy to identify CD45RA^+^RO^–^ naive tTregs among total Tregs from PBMCs (right). (**C**) Left: Intracellular FOXP3/Helios expression (left) and cell surface naive/memory marker expression (right) for naive tTregs (black) and CD28-costimulated (blue) and TNFR2-costimulated (red) tTregs. (**D**) Top: Representative plots showing expression of Th lineage–associated markers (**A**) on naive, CD28-costimulated, or TNFR2-costimulated tTregs. Bottom: Positive controls showing expression of the analyzed markers on indicated cell populations, as measured directly ex vivo. (**E**) Representative assessment of the capacity of CD28- or TNFR2-costimulated tTregs to produce Th lineage–associated cytokines after PMA/ionomycin-based restimulation (top), with positive staining controls as indicated (bottom). (**B**–**E**) Data are representative of *n* = 5.

Depending on cytokine input, activated CD4^+^ Tconvs can differentiate into different Th cell subsets based on induction of lineage-specific transcription factors (Figure 5A). Within NLT, Tregs can adapt their differentiation state in response to locally produced cytokines and, for instance, produce Th-type cytokines (6, 42). We determined by spectral flow cytometry whether naive tTregs undergo Th-like specialization in vitro upon CD3-mediated activation and costimulation via CD28 or TNFR2. For this analysis, our starting population of naive tTregs was gated on a CD25^hi^CD127^lo^CD45RA^+^CD45RO^–^ phenotype (Figure 5B). Naive tTregs were FOXP3^+^Helios^+^ and CD45RA^+^ and, after 7 days of culture, had upregulated FOXP3 and Helios expression and acquired a CD45RO^+^ effector phenotype (Figure 5C). tTregs did not acquire lineage-determining transcription factors BCL6, T-bet, or RORγt under any culture condition, while controls validated the mAbs used (Figure 5D). GATA3 was induced in activated tTregs, especially following TNFR2 costimulation (Figure 5D). Of all chemokine receptors tested, CXCR3 was particularly induced upon CD28 costimulation, while CCR4 was induced following either CD28 or TNFR2 costimulation (Figure 5D). Effector cytokine production characteristic of Th cell subsets was analyzed after PMA/ionomycin-based restimulation. Activated tTregs produced TNF, as reported previously (43), regardless of the type of costimulation. However, they did not produce any of the Th-specific cytokines IL-21, IFN-γ, IL-17A, IL-4, or IL-13 (Figure 5E). Thus, tTregs did not undergo Th-type lineage differentiation during in vitro stimulation, regardless of the costimulus used. Expression of chemokine receptors CXCR3 and CCR4 is in line with effector differentiation (2), and GATA3 expression is likewise a feature of all effector Tregs, sustaining their suppressive function and their accumulation at inflamed sites (44, 45).

### TNFR2-costimulated tTregs resemble Tregs residing in healthy NLT.

**Figure 6:**
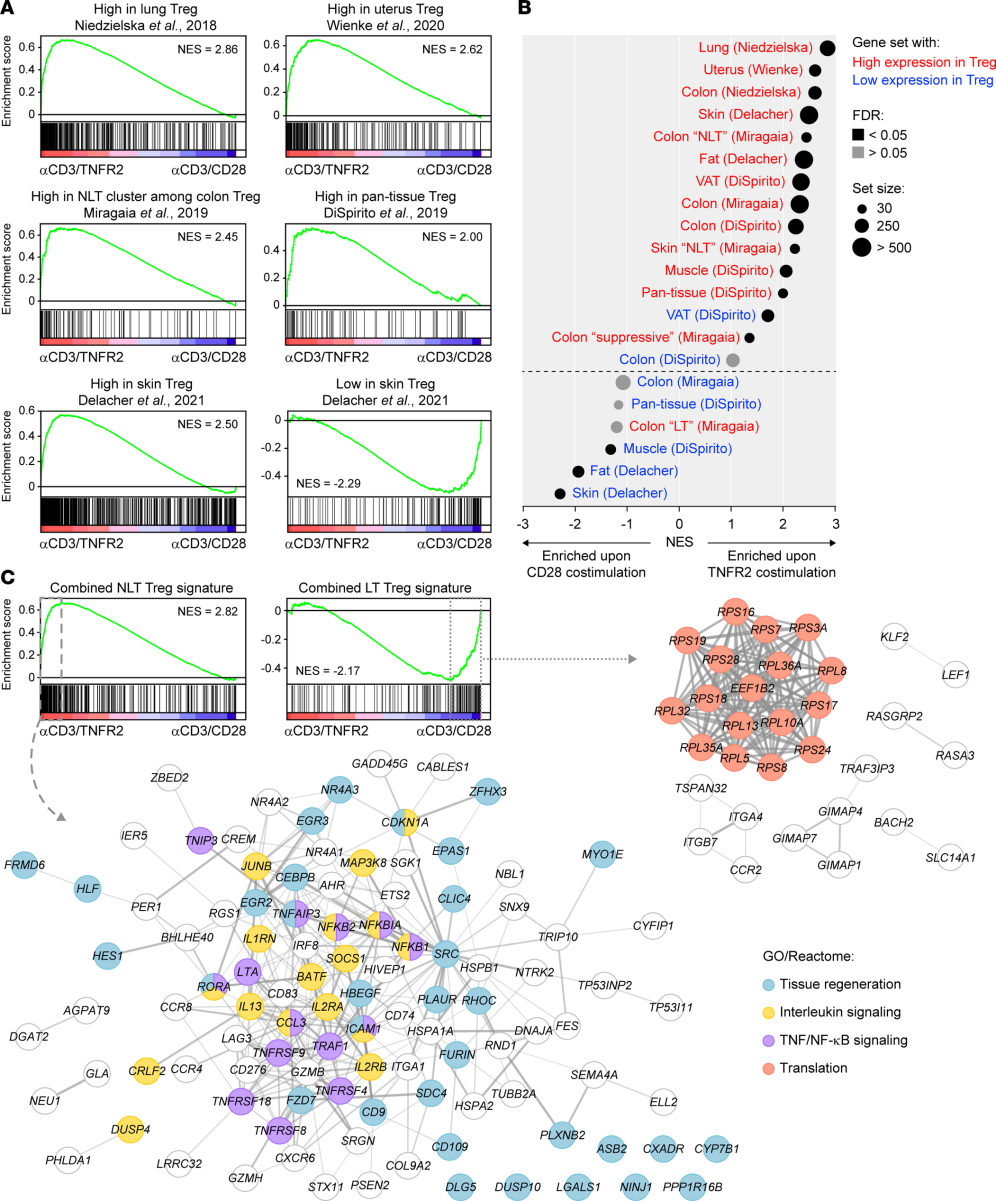
Enrichment of healthy NLT Treg gene signatures in TNFR2-costimulated tTregs. (**A**) GSEA plots showing enrichment of indicated published Treg gene signatures from healthy tissues and peripheral blood (40, 46–49) in the transcriptomes of tTregs costimulated via TNFR2 or CD28 for 7 days. Normalized enrichment scores (NES) are depicted (FDR < 0.05). (**B**) Summary of GSEA data showing enrichment of published Treg gene signatures in healthy tissues (40, 46–49) in the transcriptome of TNFR2- or CD28-costimulated tTregs. Red and blue text mark gene sets describing, respectively, high and low expression in Tregs from indicated tissues. The first author of the corresponding reference is shown between parentheses. Additional information is described in Supplemental Table 2. VAT, visceral adipose tissue. (**C**) NLT-related gene sets were compared to find overlap, leading to a combined NLT Treg gene signature composed of genes that were present in at least 3 of 9 selected NLT-related gene sets (40, 46–49). Similarly, a combined LT Treg gene signature consisted of genes present in at least 2 of 5 selected LT-related gene sets. The selection of gene sets is shown in Supplemental Table 2. GSEA was performed using the combined NLT and LT Treg signatures, which is displayed together with NES (FDR < 0.05). Leading edge genes are marked by dashed or dotted lines and visualized as STRING networks, including enriched biological processes according to GO/Reactome. Disconnected nodes not associated with labeled processes are not shown.

Tregs reside in LT and NLT, where they have different phenotypes and functions (5). To examine how tTregs costimulated via TNFR2 or CD28 relate to Tregs found in vivo, we extracted gene signatures from published transcriptomics data. In the reported studies, NLT-resident Treg signatures were derived from transcriptomic comparison of Tregs from healthy NLT versus LT and peripheral blood. The gene signatures cover Tregs from the human lung (46), colon (46), skin (40), s.c. fat (40) and uterus (47), as well as the murine colon (48, 49), visceral adipose tissue (49), and skeletal muscle (49). Using GSEA, we found that highly expressed signature genes of NLT-resident Tregs were enriched in TNFR2-costimulated tTregs, as compared with CD28-costimulated tTregs. For example, genes that were highly expressed in CD45RO^+^ effector Tregs from human lung, as compared with CD45RO^+^ Tregs from peripheral blood according to Niedzielska et al. (46), were strongly enriched in TNFR2- as opposed to CD28-costimulated tTregs (Figure 6, A and B). In a study by Miragaia et al., Tregs from murine colon and skin were discerned based on single-cell RNA-Seq (scRNA-Seq) as LT-like, NLT-like, and suppressive (48). Gene signatures of the NLT-like and suppressive Tregs were specifically enriched in TNFR2-costimulated tTregs (Figure 6, A and B). Conversely, the LT-like Treg signature was enriched in CD28-costimulated tTregs (Figure 6B).

To determine how NLT- or LT-resident Tregs differed from one another, we generated a combined NLT Treg signature comprising 452 genes present in at least 3 of 9 selected NLT gene sets, and a combined LT Treg signature including 245 genes found in at least 2 of 5 selected LT gene sets (Supplemental Tables 2 and 3). Next, we determined which features of NLT- or LT-resident Tregs were shared with TNFR2- or CD28-costimulated tTregs. For this purpose, we performed GSEA using the combined NLT and LT Treg signatures and analyzed the leading edges, revealing the enriched TNFR2- and CD28-driven transcripts (Figure 6C). Interestingly, many of the TNFR2-driven molecules from the NLT data set were functionally interconnected, as demonstrated by STRING network analysis (Figure 6C, bottom). We had already identified a number of these molecules as TNFR2-driven in vitro (e.g., TNFRSF4, TNFRSF9, CCR8, ICAM1, LRRC32) (Figure 2 and Figure 4), but this analysis shows that TNFR2 costimulation creates a Treg phenotype that is present in vivo in a great variety of NLTs. Functional annotation of the molecules according to GO/Reactome revealed, for the TNFR2-driven gene set, confirmatory TNF/NF-κB signaling and interleukin signaling but, most interestingly, tissue regeneration. The latter function has recently been ascribed to NLT-resident Tregs (40, 50), and our data show that it can be driven by TNFR2 costimulation. Analysis of the CD28 costimulation–enriched common LT-resident Treg signature revealed 1 tightly interconnected network of molecules involved in ribosome biogenesis/protein translation, but it revealed no evidence for effector differentiation — i.e., the acquisition of specific functional properties (Figure 6C). This observation fits with the fact that CD28 costimulation helps to prepare T cells for cell division (51). In summary, in vitro TNFR2 costimulation of naive tTregs from human blood specifically drives the acquisition of a gene expression and functional profile of NLT-resident Tregs.

### TNFR2-costimulated tTregs resemble Tregs present in tumors and autoimmune lesions.

**Figure 7:**
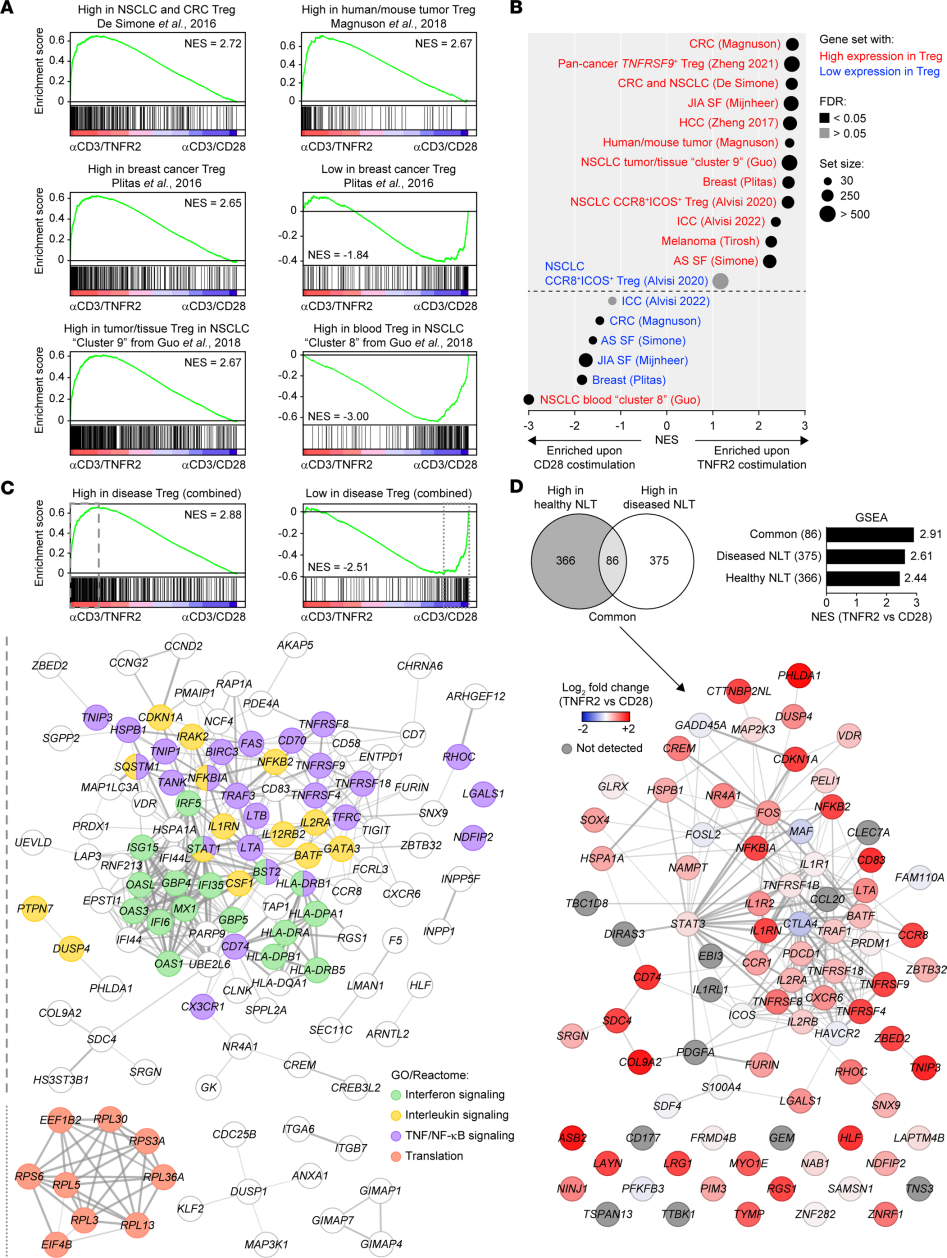
TNFR2-costimulated tTregs acquire characteristics of Tregs in tumors and autoimmune lesions. (**A**) GSEA showing enrichment of indicated published Treg gene signatures from tumors (41, 52–54) in the transcriptomes of tTregs costimulated via TNFR2 or CD28 for 7 days (FDR < 0.05). (**B**) GSEA summary showing enrichment of published Treg gene signatures from tumors and autoimmune lesions (39, 41, 52–60) in the transcriptome of TNFR2- or CD28-costimulated tTregs. Gene sets describing high or low expression in Tregs from indicated tissues are marked in red or blue, respectively. The first author of the corresponding reference is shown. Additional information is described in Supplemental Table 2. HCC, hepatocellular carcinoma; ICC, intrahepatic cholangiocarcinoma; SF, synovial fluid. (**C**) Gene sets comprising either high or low expression in Tregs in diseased NLT were compared to find overlap, generating 2 combined gene signatures. Selection of gene sets is shown in Supplemental Table 2. GSEA using the combined gene signatures is shown (FDR < 0.05). Leading edge genes are marked by dashed or dotted lines and visualized as STRING networks, including enriched biological processes according to GO/Reactome. STRING visualization of the TNFR2-enriched gene set was based on the top 150 genes of the leading edge. Disconnected nodes not associated with labeled processes are not shown. (**D**) Top left: Venn diagram showing unique and 86 overlapping highly expressed genes in Tregs in healthy (Figure 6C) versus diseased NLT. Top right: GSEA showing the NES of genes of the indicated Treg gene signatures in the transcriptomes of TNFR2- versus CD28-costimulated tTregs. Bottom: Visualization of the 86-gene common signature by STRING network analysis. Log_2_ fold changes are color coded.

Next, we determined by the same methodology whether tTregs costimulated via TNFR2 or CD28 gain features of Tregs described in diseased human tissues, using published Treg gene signatures from cancer and autoimmune diseases (Supplemental Table 2) (39, 41, 52–60). In pioneering studies, De Simone et al. and Plitas et al. compared, in patients with colorectal cancer (CRC) (52), non–small-cell lung cancer (NSCLC) (52), or breast cancer (41), the transcriptional profiles of Tregs from tumors, adjacent normal NLT, and peripheral blood. We demonstrated by GSEA that genes highly expressed in tumor-infiltrating Tregs in these studies were enriched in TNFR2-costimulated tTregs, while genes with low expression were enriched in CD28-costimulated tTregs (Figure 7, A and B). Likewise, TNFR2-costimulated tTregs were enriched for genes that were highly expressed in Tregs in human CRC or mouse tumor tissue versus normal colon tissue or spleen, respectively, according to Magnuson et al. (53) (Figure 7, A and B). Conversely, genes with low expression in Tregs in CRC tissue were enriched in CD28-costimulated tTregs (Figure 7B). More recently, a pancancer scRNA-Seq atlas of CD4^+^ and CD8^+^ T cells compiled across 21 human cancer types by Zheng et al. revealed a dominant intratumoral Treg population annotated as *TNFRSF9*^+^, which was present at lower frequencies in nonaffected NLT and even less in peripheral blood (58). We observed a specific enrichment of the *TNFRSF9*^+^ Treg signature in TNFR2-costimulated tTregs (Figure 7B), which fits with the upregulation of TNFRSF9/4-1BB by these cells (Figure 2A). An earlier scRNA-Seq study by the same group (54) described in NSCLC the signature of a Treg population (cluster 9) mostly composed of tumor-infiltrating Tregs and, to a lesser extent, of NLT Tregs; this signature was enriched in TNFR2-costimulated tTregs (Figure 7, A and B). In contrast, the signature of another Treg population (cluster 8) that was predominantly blood derived showed strong enrichment in CD28-costimulated tTregs (Figure 7, A and B).

Besides their presence in tumors, Tregs also reside at inflammatory sites in autoimmune diseases. Transcriptomic profiling of human Tregs has been reported for juvenile idiopathic arthritis (JIA) (55) and ankylosing spondylitis (AS) (56). These studies described that, compared with Tregs from peripheral blood, Tregs from synovial fluid of affected joints upregulated an effector Treg program showing similarities with tumor-infiltrating Treg gene signatures (55, 56). GSEA indicated that these synovial Treg gene signatures were enriched in TNFR2-costimulated tTregs, whereas the genes with relatively low expression were enriched in CD28-costimulated tTregs (Figure 7B).

To further understand the relationship between TNFR2- and CD28-costimulated tTregs and in vivo Tregs, we generated Treg signatures with high or low expression in NLT from cancer and autoimmune disease lesions. The Treg signature with high expression in diseased NLT comprised 461 genes present in at least 3 of 12 selected gene sets, and the Treg signature with low expression in diseased NLT comprised 142 genes found in at least 2 of 7 selected gene sets (Supplemental Tables 2 and 3). Genes with common high expression in Tregs from diseased NLT were enriched after TNFR2 costimulation (Figure 7C, top left). The leading edge genes formed a network according to STRING analysis, and functional annotation identified TNF/NF-κB and interleukin signaling as in healthy NLT, but also IFN signaling, which included MHC class II transcripts (Figure 7C). These data confirm TNFR2 as potential driver of these functions and suggest a more “alarmed,” IFN-driven state of Tregs in diseased as compared with healthy NLT. The genes with low expression in Tregs from diseased NLT were enriched in CD28-costimulated Tregs and largely formed a network with the functional annotation of protein translation (Figure 7C), as seen before in the analysis of healthy tissues.

Overall, the genes with high expression in the combined healthy or diseased NLT Treg signatures were largely distinct, suggesting that Tregs adapt to a state of disease (Figure 7D). However, there were 86 transcripts shared between these 2 conditions that we named the “common” signature of NLT-resident Tregs in health and disease. This common NLT signature showed the highest specific enrichment in TNFR2-costimulated Tregs, but overall NLT Treg signatures in both health and disease were selectively enriched in TNFR2- as compared with CD28-costimulated Tregs (Figure 7D). The common signature genes partially formed a functionally interconnected network, including diverse TNFRSF members and downstream signaling components, chemokine receptors, adhesion molecules, and diverse transcription factors, including BATF and STAT3 (Figure 7D).

### The TNFR2-driven effector tTreg phenotype is present at the protein level in the human placenta.

**Figure 8:**
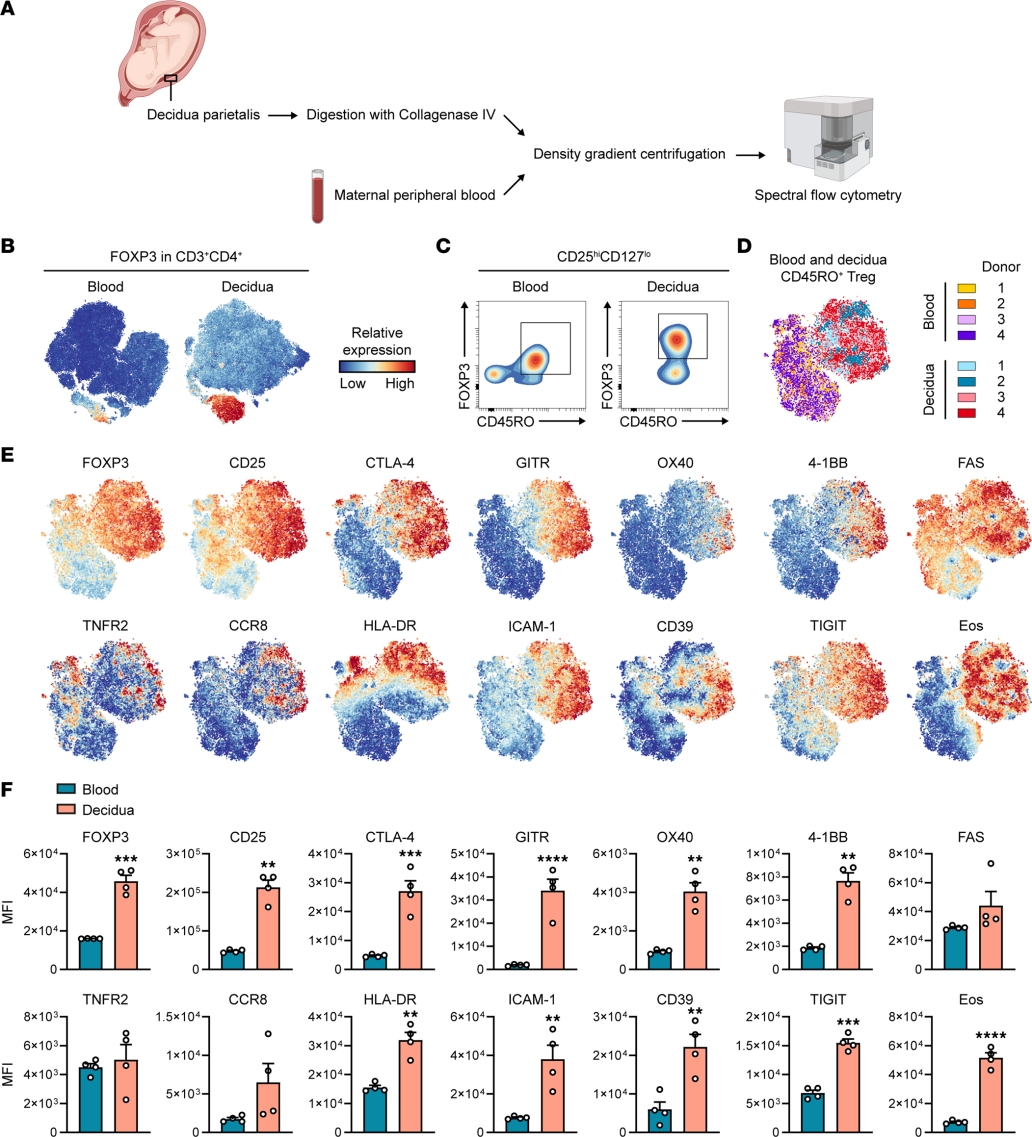
The TNFR2-driven tTreg phenotype is present at the protein level in human placenta. (**A**) Scheme depicting isolation of lymphocytes from decidua parietalis and peripheral blood of women with term pregnancies (*n* = 4). All samples were simultaneously analyzed by spectral flow cytometry. (**B**) Opt-SNE was performed on the CD3^+^CD4^+^ T cell compartment from either tissue (*n* = 4). Each dot represents a single cell. Relative protein expression of FOXP3 is color coded. (**C**) Gating strategy to identify Tregs for comparative analysis between decidua and blood. Among CD25^hi^CD127^lo^ cells, FOXP3^+^CD45RO^+^ effector Tregs were selected for further analysis. The complete gating strategy is shown in Supplemental Figure 5. (**D**) Opt-SNE was performed on effector Tregs from decidua and blood collectively, showing individual samples that are color coded. (**E**) Protein expression of indicated molecules after opt-SNE described in **D**. Relative expression is shown according to the color legend in **B**. (**F**) Quantification of protein expression in **E** as MFI, comparing decidua and blood samples (*n* = 4). Statistical analysis was done by paired 2-tailed Student’s *t* test. Data are presented as mean ± SEM. Sample size (*n*) represents individual donors. **P < 0.01, ***P < 0.001, ****P < 0.0001.

We next determined whether the TNFR2-driven tTreg phenotype can be identified in human tissue by flow cytometry. From the leading edge analyses on NLT Tregs, we selected cell surface proteins that were of functional interest and to which well-validated antibodies were available for simultaneous analysis by spectral flow cytometry. We selected CD25 (IL2RA), OX40 (TNFRSF4/CD134), 4-1BB (TNFRSF9/CD137), FAS (TNFRSF6/CD95), GITR (TNFRSF18/CD357), CCR8, HLA-DR, ICAM-1 (CD54), CD39 (ENTPD1), and TIGIT as markers for flow cytometry. We also included FOXP3, Eos, and CTLA-4 as Treg markers, as well as TNFR2 (TNFRSF1B). We selected the human placenta as NLT of interest, where Tregs are essential for a healthy pregnancy (61). By digestion and density gradient centrifugation, we isolated lymphocytes from the decidua parietalis from 4 women with term pregnancies (Figure 8A and Supplemental Table 4). We also isolated PBMCs from matched maternal peripheral blood for comparison. Spectral flow cytometry and visualization of the data by opt-SNE, a modified version of t-SNE, revealed a FOXP3^+^ cluster among CD3^+^CD4^+^ T cells from either decidua or blood (Figure 8B). To exclude Tconvs, we gated on CD3^+^CD4^+^CD25^hi^CD127^lo^ cells and next on FOXP3^+^CD45RO^+^ effector Tregs, to fairly compare effector Treg phenotypes in decidua and blood (Figure 8C and Supplemental Figure 5, A and B).

Opt-SNE visualization of decidual and blood effector Tregs combined indicated a clear separation by tissue origin (Figure 8D). Treg markers FOXP3, Eos, CD25, and total CTLA-4 were more explicitly expressed by Tregs in the decidua as compared with blood (Figure 8, E and F). Furthermore, TNFRSF members GITR, OX40, and 4-1BB were almost exclusively expressed on decidual Tregs, whereas FAS was expressed on both decidual and blood Tregs. TNFR2^+^ cells were detected in both decidua and blood, resulting in similar mean expression levels. CCR8 appeared to be predominantly expressed on decidual Tregs, albeit not significantly due to donor variability. Lastly, decidual Tregs expressed higher mean levels of HLA-DR, ICAM-1, CD39, and TIGIT than blood Tregs. In short, these data show that, with exception of FAS, all TNFR2-driven cell surface proteins and transcription factors analyzed help to discriminate effector Tregs in NLT from those in blood.

## Discussion

Clinical Treg products are generally made by stimulating Tregs purified from human blood with agonistic antibodies to CD3 and CD28. However, TNFR2 costimulation also supports CD3-driven proliferation, survival, and metabolism of human Tregs (16, 22, 30). We demonstrate here that TNFR2 and CD28 costimulation have differential effects on tTreg functionality; TNFR2 signals drive differentiation of human blood–derived tTregs into an in vivo NLT-resident phenotype, while CD28 signals maintain an in vivo LT-resident phenotype. TNFR2 and CD28 activate different signal transduction pathways, explaining their differential effect on gene expression. TNFR2 signals via TRAF adaptors and activates serine-threonine kinases (15), whereas CD28 signals analogous to CD3 via tyrosine kinases (51). In general, TNFRSF members activate NF-κB signaling pathways that are important mediators of cell survival. We observed that TNFR2-costimulated tTregs upregulate many genes involved in TNFRSF and NF-κB signaling, in agreement with a microarray-based study on the response of blood-derived Tregs to TNF (62). Soluble TNF that can bind to both TNFR1 and TNFR2 had similar effects on tTreg proliferation and expression of specific proteins as agonistic mAb to TNFR2. These effects are therefore likely mediated by TNFR2 that has a much higher expression level on tTregs than TNFR1 (13, 16, 18).

We identified TNF/NF-κB signaling and cell survival as common pathways activated in TNFR2-costimulated tTregs in vitro and NLT-resident Tregs in vivo. Potential for long-term survival is an attractive feature for NLT-resident Tregs. Next to TNFR2, other TNFRSF members may contribute to survival signaling in NLT-resident Tregs following their upregulation upon TNFR2 costimulation. Particularly, OX40, 4-1BB, and GITR were upregulated on tTregs by TNFR2 costimulation and had higher cell surface expression on Tregs from NLT, according to our analysis of the placenta. Costimulation via 4-1BB and GITR was found to promote murine Treg proliferation, survival, and in vivo function (63). Furthermore, TNFRSF signaling via the NF-κB transcription factor *RelA* proved important for the differentiation and maintenance of effector Tregs in mouse NLT (64).

Recent transcriptome- and TCR-based trajectory analyses show that NLT-resident Tregs are likely primed in the spleen or draining lymph nodes (40, 48, 65). These data argue that, following priming in LT, Tregs become differentiated effector/memory-like cells in NLT (40, 48, 58, 65, 66). A recent systematic analysis showed that Tregs from a variety of NLT share gene expression profiles (67). Tregs from different NLT also shared TCR sequences and populated different NLT upon adoptive transfer, regardless of their tissue of origin (67). NLT-resident Tregs apparently share a common gene expression program but may also undergo unique tissue-specific adaptations upon NLT entry (48). For example, local cytokine milieus may provide further input for the described Th-like Treg polarization (42). TNFR2 costimulation instructed a general NLT gene expression program in blood-derived naive tTregs, without Th-like polarization, supporting the concept that NLT-resident Tregs originate from tTregs in LT. Thus far, it is unknown whether TNFR2 drives this differentiation pathway in vivo and which TNFR2 ligand triggers it. However, it is likely that TNF–TNFR2 signaling is important for Tregs both in LT and NLT, based on multiple findings. Firstly, Treg-specific TNFR2 deficiency compromises Treg function in spleen and lymph nodes (17). Also, Treg activation in lymph nodes partially depends on TNF produced by Tconvs (68). Furthermore, Tregs need TNFR2 to remain stable and functional in inflammatory environments and to control autoimmune diseases (17, 18, 21).

Tregs can find TNF on Tconvs (20, 68), Tregs (43), monocytes (69), and tolerogenic monocyte-derived DCs (70). TNFR2 may help Tregs to control inflammation by sequestering TNF and attenuating TNFR1-driven inflammation, as well as by promoting Treg expansion and differentiation into NLT-resident effector cells. Increased local TNF levels in tumors and diseased synovial fluid may drive the explicit effector phenotypes of Tregs observed in these tissues (41). Besides TNF, lymphotoxin α_3_ (LTα_3_) and LTα_2_β_1_ are TNFR2 ligands (14). Lymphotoxins are expressed by various lymphoid and myeloid cells and may also drive TNFR2 costimulation of Tregs. LTα_1_β_2_ expression by Tregs is reportedly required for their stability and migration across LTβR-expressing endothelial cells (71). We observed *LTA* and *LTB* in the transcriptomic signature of NLT-resident Tregs, suggesting that they produce these ligands themselves.

TNFR2-costimulated tTregs and NLT-resident Tregs express multiple transcription factors that may dictate their functional abilities. Among those, BATF is known to drive the stepwise reprogramming of Tregs found in LT toward the most differentiated Tregs found in healthy NLT and tumors (40, 66, 72). Effector Treg differentiation is also regulated by Blimp-1, encoded by *PRDM1* (73). Both TNFR2- and CD28-costimulated tTregs expressed higher mRNA levels of *BATF* and *PRDM1* than their Tconv counterparts (data not shown). Myb is another transcription factor crucial for effector differentiation of tTregs (74). We found *MYB* upregulation following TNFR2 costimulation, in line with reported findings in WT versus TNFR2-deficient Tregs (21). Moreover, we observed that TNFR2-costimulated tTregs have increased levels of *NR4A3*, which together with NR4A1 and NR4A2 maintains Treg stability by controlling FOXP3 expression (75). We also found that TNFR2 costimulation upregulates GATA3 in tTregs. This transcription factor is known for its role in Th2-type Tconv differentiation but controls FOXP3 expression in Tregs, pairs with FOXP3, and is generally important for effector Treg homeostasis (44, 45, 76).

We found that TNFR2-costimulated tTregs upregulate molecules involved in migration, adhesion, and suppressive function. These functions may be highly relevant for Tregs to reach and operate in NLT. Adhesion molecules ICAM-1 and LAYN are highly expressed on Tregs in both healthy and diseased NLT, as well as chemokine receptor CCR8 (40, 41). We confirmed by functional studies that, compared with CD28 costimulation, TNFR2 costimulation specifically increased migratory activity of tTregs, including chemotaxis toward CCR8 ligand CCL1. CCR8 is highly expressed on NLT-resident Tregs, including those in tumors, and is considered a suitable target for tumor-infiltrating Treg depletion (77). In addition to facilitating migration, CCR8 can promote proliferation and suppressive function of Tregs via autocrine CCL1/CCR8 signaling that also increases CCR8 expression (78).

Migratory capacities of Tregs are connected to cell metabolism. Treg migration to inflammatory sites was shown to rely on glucokinase-mediated glycolysis induced by the PI3K/mTORC2 pathway, leading to cytoskeletal rearrangements (79). Glycolysis provides glucose-derived carbon for biosynthesis of nucleotides, amino acids, and lipids that are required for cell proliferation, effector differentiation, and function (80). We recently described that TNFR2 costimulation promotes a glycolytic switch in tTregs via PI3K and mTOR, which is important for their identity and suppressive function (22). We propose that TNFR2-induced glycolysis in tTregs is tightly connected to their differentiation into NLT-resident Tregs and the gain of migratory properties.

Our findings underline the importance of TNFR2 as a target for the clinical modulation and use of Tregs. Treatment of autoimmune and chronic inflammatory diseases commonly involves TNF-blocking agents to inhibit the proinflammatory effects of TNFR1 signaling (13). However, these drugs are ineffective for part of the patients, which may be explained by concomitant inhibition of antiinflammatory properties of TNF, likely mediated by TNFR2 signaling in Tregs (13, 15). Selective TNFR1 inhibition or TNFR2 agonism may therefore be more suitable approaches for antibody-based therapy. TNFR2 agonism to combat autoimmunity and inflammation is currently under development, while TNFR2 antagonism might be used to increase antitumor immunity (19, 81). Adoptive Treg therapy may benefit from generation of Treg products with NLT-specific characteristics, such as homing properties, for the tailored treatment of organ-specific inflammatory diseases. In conclusion, our study illuminates that TNFR2 costimulation installs characteristics of NLT-resident Tregs in human blood–derived naive tTregs, including features that promote cell survival, migration, immunosuppression, and tissue regeneration. Therefore, our findings present TNFR2 as an attractive target to drive Treg differentiation for therapeutic use.

## Methods

### Sex as biological variable.

Our study examined cells from male and female patients. Sex was not considered as a biological variable.

### Isolation and culture of human blood–derived T cells.

Human CD4^+^ T cells were isolated from buffy coats from healthy donors aged 18–65 years using the StraightFrom Buffy Coat CD4 MicroBead kit (Miltenyi Biotec) according to the manufacturer’s instructions. For cell sorting, CD4^+^ T cells were stained with CD4-PE-Cy7 (317414, BioLegend), CD127-BV421 (351310, BioLegend), CD25-PE (341011, BD Biosciences), CD45RA-FITC (21819453, ImmunoTools), and GPA33-AF647 mAbs. Anti-GPA33 mAb was provided by D. Amsen (Sanquin, Amsterdam, Netherlands) and conjugated in-house to Alexa Fluor 647 succinimidyl ester (Invitrogen) as described (24). Cell viability was assessed with LIVE/DEAD near-IR dye (Invitrogen). Naive Tconvs and tTregs were sorted based on CD4^+^CD25^lo^CD127^hi^CD45RA^+^GPA33^int^ and CD4^+^CD25^hi^CD127^lo^CD45RA^+^GPA33^hi^ phenotypes, respectively, using a BD FACSAria II sorter with FACSDiva software (version 8, BD Biosciences) (Supplemental Figure 1A).

After cell sorting (day 0), naive Tconvs and tTregs were cultured for 7 days at 37°C/5% CO_2_ in 96-well round bottom plates (Thermo Fisher Scientific; 1 × 10^4^ cells per well) in 100 μL IMDM (Capricorn Scientific) with 8% FCS (Serana), penicillin/streptomycin (Thermo Fisher Scientific), and IL-2 (300 IU/mL, DuPont Medical). Soluble agonistic mAb to CD3 (0.1 μg/mL, clone CLB-T3/4.E, IgE isotype, Sanquin) was used for TCR/CD3 stimulation, and soluble agonistic mAb to either CD28 (0.2 μg/mL, clone CLB-CD28/1, Sanquin) or TNFR2 (2.5 μg/mL, clone MR2-1, Hycult Biotech) was used for costimulation. Recombinant human TNF (PeproTech) was used incidentally, as indicated. On day 4, 100 μL supplemented IMDM including IL-2 (300 IU/mL) was added to the culture (Supplemental Figure 1B).

### Proliferation assay.

For proliferation assays based on dye dilution, naive Tconvs and tTregs were washed in PBS and labeled for 8 minutes with 5 μM CTV (Invitrogen) at 37°C. Next, an equal volume of FCS was added, followed by 5-minute incubation, and cells were washed twice in supplemented IMDM. CTV-labeled T cells (1 × 10^4^ cells per well) were cultured for 96 hours in the presence of IL-2 (300 IU/mL) and indicated agonistic mAbs at 37°C/5% CO_2_. Proliferation was assessed by flow cytometry on a BD FACS Canto II or LSR II analyzer with FACSDiva software (version 8, BD Biosciences). Dead cells were excluded with LIVE/DEAD near-IR dye. Alternatively, proliferation was assessed based on cell counting with Precision Count Beads (BioLegend) according to the manufacturer’s instructions. Data were analyzed using FlowJo software (version 10.8.1).

### Suppression assay.

PBMCs were washed once in PBS and labeled for 8 minutes with 5 μM CTV (Invitrogen) at 37°C. After addition of an equal volume of FCS and 5-minute incubation, cells were washed twice in supplemented IMDM. CTV-labeled PBMCs were cocultured at indicated ratios with tTregs in 96-well round-bottom plates (Thermo Fisher Scientific), in the presence of soluble anti-CD3 agonistic mAb (0.05 μg/mL). The tTregs had been costimulated for 7 days and cultured for 4 additional days without agonistic mAbs in fresh supplemented IMDM with IL-2 (300 IU/mL). After 4 days of coculture of PBMCs and tTregs, the cells were stained with CD3-PE (R0810, Dako), CD4-FITC (21810043, ImmunoTools), and CD8-APC (21810086, ImmunoTools) mAbs and viability was determined with LIVE/DEAD near-IR dye. Proliferation of CTV-labeled CD4^+^ and CD8^+^ T cells was analyzed by flow cytometry using a BD LSR Fortessa or BD LSR II cell analyzer, and data were analyzed using FlowJo software.

### Migration assays.

For transwell migration assays, 6.5 mm diameter Transwell inserts (5 μm pore, polycarbonate membrane, Corning) were coated with recombinant protein G (20 μg/mL, Sigma-Aldrich), washed with PBS, coated with recombinant human ICAM-1-Fc (5 μg/mL, BioLegend), and washed with PBS/0.5% FCS. Costimulated tTregs were harvested on day 7 and washed in IMDM/0.5% FCS. Next, 100,000 cells were placed on top of the inserts in 100 μL IMDM/0.5% FCS with IL-2 (300 IU/mL). After 45 minutes, inserts were placed in a 24-well plate, containing 600 μL IMDM/0.5% FCS with IL-2 and either no chemokine, or CCL1 (200 ng/mL, R&D Systems), or CXCL12 (100 ng/mL, PeproTech). In separate input control samples, 100,000 cells were cultured in the bottom chamber. Cells were incubated at 37°C/5% CO_2_ for 16 hours and counted with Precision Count Beads by flow cytometry, using a BD LSR II cell analyzer and FlowJo software. The frequency of migrated cells was calculated as a percentage of the input control.

For kinetic analysis of cell migration, inserts of an Incucyte Clearview 96-well plate for chemotaxis (Sartorius) were coated as described above, in duplicate. Next, 5,000 washed tTregs were placed on top of the inserts in 60 μL IMDM/0.5% FCS with IL-2 (300 IU/mL). After 45 minutes, inserts were placed in the 96-well plate, the wells of which contained 200 μL IMDM/0.5% FCS including IL-2 and either no chemokine, with CCL1 (200 ng/mL), or with CXCL12 (100 ng/mL). Cells were incubated in an Incucyte S3 live-cell analysis system (Sartorius) at 37°C/5% CO_2_ for 16 hours, with hourly scans. Migration was quantified by measuring the area occupied by cells on top of the insert normalized to the area at t = 0 hours, using the Incucyte chemotaxis analysis software module (Sartorius).

### Isolation of lymphocytes from human placenta and maternal peripheral blood.

Third-trimester placentas were collected after spontaneous delivery or cesarean section. Matched maternal peripheral blood was obtained at a gestational age of 36 weeks or at delivery. Clinical characteristics of the patients included are summarized in Supplemental Table 4. Lymphocytes were isolated from human decidua parietalis according to a protocol adapted from Tilburgs et al. (82). Briefly, decidua parietalis was macroscopically dissected by removing the amnion and by delicately scraping it off of the chorion. Next, tissue was washed in PBS, minced, and resuspended in 0.2 mg/mL Collagenase IV (MilliporeSigma) and 0.01 mg/mL DNAse I (MilliporeSigma) dissolved in RPMI 1640 (Life Technologies) at a ratio of 10 mL digestion mix per 5 g tissue. Tissue was homogenized with a GentleMACS dissociator (Miltenyi Biotec), and samples were passed through 250 μm and 70 μm filters sequentially and washed in RPMI. Cell suspensions were mixed with 20 mL of 1.023 g/mL Percoll (GE Healthcare) and separated by density gradient centrifugation (820*g*, no brake, room temperature, 30 minutes) on a Percoll gradient (10 mL of 1.080 g/mL, 15 mL of 1.053 g/mL). Lymphocytes were isolated from the 1.080–1.053 g/mL interface and washed twice in RPMI. PBMCs were isolated from maternal blood by Ficoll-Paque (GE Healthcare) density gradient centrifugation (820*g*, no brake, room temperature, 20 minutes). All samples were frozen and stored in liquid nitrogen until use.

### Phenotypic analysis by flow cytometry.

To measure cell surface protein expression, cells were washed in PBS/1% FCS, incubated with human Fc-Block (BioLegend) for 5 minutes on ice, and stained for 30 minutes on ice with mAbs in appropriate combinations. Cell viability was assessed with LIVE/DEAD near-IR or blue dye (Invitrogen). Chemokine receptor-specific mAbs were incubated for 20 minutes at 37°C instead of on ice. To measure cytokine production capacity, costimulated tTregs were treated with the eBioscience cell stimulation cocktail including protein transport inhibitors (Invitrogen), based on PMA, ionomycin, brefeldin A, and monensin. For intracellular staining, cells were fixed and permeabilized using the FOXP3 transcription factor staining buffer set (Invitrogen) according to the manufacturer’s instructions. Cells were stained for 30–45 minutes on ice using mAbs in appropriate combinations. After washing, flow cytometry was performed on a BD LSR Fortessa or BD LSR II analyzer with FACSDiva software. Data were analyzed using FlowJo software. All antibodies used in this study are listed in Supplemental Table 5.

Regarding Figure 5, positive controls to validate mAbs included healthy donor–derived PBMCs, human tonsils, and Th2-polarized Tconvs. Briefly, tonsils were minced and mechanically disrupted with a syringe plunger and a stainless steel sieve. Cells were passed through a 70 μm cell strainer, and mononuclear cells were isolated by Ficoll-Paque density gradient centrifugation (820*g*, no brake, room temperature, 20 minutes). Th2 cell polarization was performed by culturing naive Tconvs until day 7 as described, in the presence of recombinant human IL-4 (10 ng/mL, BioLegend) and anti–human IFN-γ blocking mAb (1 μg/mL, BioLegend).

For LRRC32 detection (Figure 2D), and the data depicted in Figures 5 and 8, samples were analyzed by spectral flow cytometry using a 5-laser Cytek Aurora with SpectroFlo software (Cytek Biosciences). Data were analyzed using FlowJo software (Figure 2D and Figure 5) or the OMIQ platform (https://www.omiq.ai/) (Figure 8). In OMIQ, the FlowAI algorithm was used for anomaly detection and exclusion, followed by data scaling and compensation when necessary. After gating (Supplemental Figure 5, A and B), dimension reduction was performed by opt-SNE including FOXP3, CD25, CTLA-4, GITR, OX40, 4-1BB, FAS, TNFR2, CCR8, HLA-DR, ICAM-1, CD39, TIGIT, Eos, CD127, CD45RA, CD45RO, and Ki-67. Mean expression data were exported for quantification.

### Transcriptome analysis.

CD28- or TNFR2-costimulated Tconvs and tTregs (1 × 10^5^ per sample) were collected on day 7 of cell culture and subjected to bulk transcriptomics, as detailed in the Supplemental Methods. BAM files were imported in Qlucore Omics Explorer software (version 3.8) with GRCh38.104.gtf as a reference genome, using trimmed mean of M values (TMM) normalization (83). Transcripts with less than 10 reads in at least 80% of the samples were excluded from downstream expression analysis, as were remaining transcripts with low expression in all samples. PCA was used to visualize the data in 3-dimensional space. Differential gene expression between 2 groups was determined by a Student’s *t* test with the Benjamini-Hochberg method for multiple testing correction and considered significant at FDR < 0.05. Heatmaps with *Z* score normalization and hierarchical clustering were created using Qlucore Omics Explorer. Volcano plots were created using GraphPad Prism (version 9.3.1).

Differentially expressed genes were analyzed for associated biological processes using Metascape with default parameters (enrichment factor > 1.5, *P* < 0.01) (84). Overrepresented GO/Reactome terms were visualized using Cytoscape software (version 3.10.1). Alternatively, analysis of differentially regulated cellular functions was performed using IPA software (version 84978992, QIAGEN) with the right-tailed Fisher’s exact test and the Benjamini-Hochberg method (FDR < 0.05). Interaction networks of genes or proteins, including associated GO/Reactome terms, were generated using the STRING app (version 2.0.1) within Cytoscape.

GSEA software (version 4.3.2) was used to analyze whether defined gene sets were enriched in comparative experimental conditions. GSEA was performed using the Hallmark gene set collection from the Molecular Signatures Database (https://www.gsea-msigdb.org/gsea/index.jsp) or published gene sets as detailed in Supplemental Table 2. Mouse genes were converted to human orthologs using Ensembl BioMart for comparison with the human transcriptomics data in the present study. In addition, GSEA was performed using differentially expressed genes or proteins from transcriptome (22, 23) or proteome analyses, respectively, as indicated (Figure 3D and Supplemental Figure 4E).

### Proteomics.

Naive tTregs were expanded for 7 days using CD28 costimulation, as described above, and were cultured for 4 additional days in the absence of agonistic mAbs and in the presence of IL-2. Next, 1 × 10^6^ cells were cultured for 24 hours in 24-well plates, in the presence of IL-2, anti-CD3 mAb, and either anti-CD28 or anti-TNFR2 mAb. Restimulated cells were collected for proteomics, as detailed in the Supplemental Methods.

### Statistics.

Data, excluding those describing transcriptomics or proteomics data, were analyzed using GraphPad Prism (version 9.3.1). Statistical analyses were performed as indicated in the figure legends. Data were log-transformed in case data were not normally distributed. Data are represented as mean ± SEM. A 2-sided *P* < 0.05 was considered statistically significant.

### Study approval.

Human peripheral blood was obtained in agreement with the Declaration of Helsinki and the Dutch rules regarding the use of human materials from volunteer donors. Buffy coats were obtained from healthy, anonymized donors aged 18–65 with written informed consent as approved by the Ethical Advisory Council of Sanquin Blood Supply Foundation (License NVT0111.01; Amsterdam, Netherlands). Tonsil samples were from routine tonsillectomies collected at the Alrijne Hospital (Leiderdorp, Netherlands) with written informed consent and approved by the Medical Ethics Committee of Leiden University Medical Center (protocol B21.019). Human placenta and maternal peripheral blood samples were obtained after written informed consent, and the study was carried out in accordance with the guidelines issued by the Medical Ethics Committee of Leiden University Medical Center (protocol P16.048) and in accordance with the Declaration of Helsinki.

### Data availability.

Transcriptomics data derived from cells that were cultured for 7 days (GSE253540), or from preexpanded cells that were restimulated for 24 hours (22, 23) (GSE138604), are publicly accessible in the GEO database. Proteomics data derived from preexpanded cells that were restimulated for 24 hours have been deposited to the ProteomeXchange Consortium via the PRIDE repository with the data set identifier PXD048248 (https://proteomecentral.proteomexchange.org/). Individual data values presented in graphs can be accessed in the Supporting Data Values file.

## Author contributions

MM designed, performed, and analyzed experiments and prepared the manuscript. LJV designed, performed, and analyzed experiments presented in Figure 8. ES provided technical support. GMCJ, RTNT, and PVV performed mass spectrometry and proteome analysis. QJ and MFP provided mAbs and positive control samples for Th differentiation analysis. MLVDH and ME provided human tissue samples. SDK performed experiments and provided intellectual input. JB designed the study, supervised the project, and prepared the manuscript. All authors contributed to and approved of the manuscript.

## Supplementary Material

Supplemental data

Supplemental table 1

Supplemental table 2

Supplemental table 3

Supplemental table 4

Supplemental table 5

Supporting data values

## Version 1 01/30/2024

In-Press Preview

## Version 2 03/08/2024

Electronic publication

## References

[1] Ohkura N (2013). Development and maintenance of regulatory T cells. Immunity.

[2] Opstelten R, Amsen D (2021). Separating the wheat from the chaff: Making sense of Treg heterogeneity for better adoptive cellular therapy. Immunol Lett.

[3] Josefowicz SZ (2012). Extrathymically generated regulatory T cells control mucosal TH2 inflammation. Nature.

[4] Samstein RM (2012). Extrathymic generation of regulatory T cells in placental mammals mitigates maternal-fetal conflict. Cell.

[5] Liston A, Gray DH (2014). Homeostatic control of regulatory T cell diversity. Nat Rev Immunol.

[6] Josefowicz SZ (2012). Regulatory T cells: mechanisms of differentiation and function. Annu Rev Immunol.

[7] Tanaka A, Sakaguchi S (2017). Regulatory T cells in cancer immunotherapy. Cell Res.

[8] Hippen KL (2022). Emerging translational strategies and challenges for enhancing regulatory T cell therapy for graft-versus-host disease. Front Immunol.

[9] Raffin C (2020). T_reg_ cell-based therapies: challenges and perspectives. Nat Rev Immunol.

[10] Harden PN (2021). Feasibility, long-term safety, and immune monitoring of regulatory T cell therapy in living donor kidney transplant recipients. Am J Transplant.

[11] Bluestone JA (2015). Type 1 diabetes immunotherapy using polyclonal regulatory T cells. Sci Transl Med.

[12] Desreumaux P (2012). Safety and efficacy of antigen-specific regulatory T-cell therapy for patients with refractory Crohn’s disease. Gastroenterology.

[13] Salomon BL (2021). Insights into the biology and therapeutic implications of TNF and regulatory T cells. Nat Rev Rheumatol.

[14] Kucka K (2021). Membrane lymphotoxin-α_2_β is a novel tumor necrosis factor (TNF) receptor 2 (TNFR2) agonist. Cell Death Dis.

[15] Tseng WY (2018). TNFR signalling and its clinical implications. Cytokine.

[16] Chen X (2007). Interaction of TNF with TNF receptor type 2 promotes expansion and function of mouse CD4+CD25+ T regulatory cells. J Immunol.

[17] Atretkhany KN (2018). Intrinsic TNFR2 signaling in T regulatory cells provides protection in CNS autoimmunity. Proc Natl Acad Sci U S A.

[18] Chen X (2013). TNFR2 is critical for the stabilization of the CD4+Foxp3+ regulatory T. cell phenotype in the inflammatory environment. J Immunol.

[19] Chopra M (2016). Exogenous TNFR2 activation protects from acute GvHD via host T reg cell expansion. J Exp Med.

[20] Leclerc M (2016). Control of GVHD by regulatory T cells depends on TNF produced by T cells and TNFR2 expressed by regulatory T cells. Blood.

[21] Ronin E (2021). Tissue-restricted control of established central nervous system autoimmunity by TNF receptor 2-expressing Treg cells. Proc Natl Acad Sci U S A.

[22] de Kivit S (2020). Stable human regulatory T cells switch to glycolysis following TNF receptor 2 costimulation. Nat Metab.

[23] Mensink M (2022). TNFR2 costimulation differentially impacts regulatory and conventional CD4^+^ T-cell metabolism. Front Immunol.

[24] Opstelten R (2020). GPA33: a marker to identify stable human regulatory T cells. J Immunol.

[25] Lam AJ (2022). Helios is a marker, not a driver, of human Treg stability. Eur J Immunol.

[26] Pan F (2009). Eos mediates Foxp3-dependent gene silencing in CD4+ regulatory T cells. Science.

[27] Ferraro A (2014). Interindividual variation in human T regulatory cells. Proc Natl Acad Sci U S A.

[28] Watts TH (2005). TNF/TNFR family members in costimulation of T cell responses. Annu Rev Immunol.

[29] Paulsen M, Janssen O (2011). Pro- and anti-apoptotic CD95 signaling in T cells. Cell Commun Signal.

[30] He X (2016). A TNFR2-agonist facilitates high purity expansion of human low purity Treg cells. PLoS One.

[31] Baecher-Allan C (2006). MHC class II expression identifies functionally distinct human regulatory T cells. J Immunol.

[32] Joller N (2014). Treg cells expressing the coinhibitory molecule TIGIT selectively inhibit proinflammatory Th1 and Th17 cell responses. Immunity.

[33] Stockis J (2017). Role of GARP in the activation of latent TGF-β1. Mol Biosyst.

[34] Gourdin N (2018). Autocrine adenosine regulates tumor polyfunctional CD73^+^CD4^+^ effector T cells devoid of immune checkpoints. Cancer Res.

[35] Schuler PJ (2014). Human CD4+ CD39+ regulatory T cells produce adenosine upon co-expression of surface CD73 or contact with CD73+ exosomes or CD73+ cells. Clin Exp Immunol.

[36] Donati G, Amati B (2022). MYC and therapy resistance in cancer: risks and opportunities. Mol Oncol.

[37] Walling BL, Kim M (2018). LFA-1 in T cell migration and differentiation. Front Immunol.

[38] Mehta P (2021). Layilin anchors regulatory T cells in skin. J Immunol.

[39] Zheng C (2017). Landscape of infiltrating T cells in liver cancer revealed by single-cell sequencing. Cell.

[40] Delacher M (2021). Single-cell chromatin accessibility landscape identifies tissue repair program in human regulatory T cells. Immunity.

[41] Plitas G (2016). Regulatory T cells exhibit distinct features in human breast cancer. Immunity.

[42] Kitz A, Dominguez-Villar M (2017). Molecular mechanisms underlying Th1-like Treg generation and function. Cell Mol Life Sci.

[43] Urbano PCM (2018). An autocrine TNFα-tumor necrosis factor receptor 2 loop promotes epigenetic effects inducing human Treg stability in vitro. Front Immunol.

[44] Wang Y (2011). An essential role of the transcription factor GATA-3 for the function of regulatory T cells. Immunity.

[45] Wohlfert EA (2011). GATA3 controls Foxp3^+^ regulatory T cell fate during inflammation in mice. J Clin Invest.

[46] Niedzielska M (2018). Differential gene expression in human tissue resident regulatory T cells from lung, colon, and blood. Oncotarget.

[47] Wienke J (2020). Human Tregs at the materno-fetal interface show site-specific adaptation reminiscent of tumor Tregs. JCI Insight.

[48] Miragaia RJ (2019). Single-cell transcriptomics of regulatory T cells reveals trajectories of tissue adaptation. Immunity.

[49] DiSpirito JR (2018). Molecular diversification of regulatory T cells in nonlymphoid tissues. Sci Immunol.

[50] Burzyn D (2013). A special population of regulatory T cells potentiates muscle repair. Cell.

[51] Acuto O, Michel F (2003). CD28-mediated co-stimulation: a quantitative support for TCR signalling. Nat Rev Immunol.

[52] De Simone M (2016). Transcriptional landscape of human tissue lymphocytes unveils uniqueness of tumor-infiltrating T regulatory cells. Immunity.

[53] Magnuson AM (2018). Identification and validation of a tumor-infiltrating Treg transcriptional signature conserved across species and tumor types. Proc Natl Acad Sci U S A.

[54] Guo X (2018). Global characterization of T cells in non-small-cell lung cancer by single-cell sequencing. Nat Med.

[55] Mijnheer G (2021). Conserved human effector Treg cell transcriptomic and epigenetic signature in arthritic joint inflammation. Nat Commun.

[56] Simone D (2021). Single cell analysis of spondyloarthritis regulatory T cells identifies distinct synovial gene expression patterns and clonal fates. Commun Biol.

[57] Tirosh I (2016). Dissecting the multicellular ecosystem of metastatic melanoma by single-cell RNA-Seq. Science.

[58] Zheng L (2021). Pan-cancer single-cell landscape of tumor-infiltrating T cells. Science.

[59] Alvisi G (2020). IRF4 instructs effector Treg differentiation and immune suppression in human cancer. J Clin Invest.

[60] Alvisi G (2022). Multimodal single-cell profiling of intrahepatic cholangiocarcinoma defines hyperactivated Tregs as a potential therapeutic target. J Hepatol.

[61] Robertson SA (2018). Regulatory T cells in embryo implantation and the immune response to pregnancy. J Clin Invest.

[62] Nagar M (2010). TNF activates a NF-kappaB-regulated cellular program in human CD45RA- regulatory T cells that modulates their suppressive function. J Immunol.

[63] Lubrano di Ricco M (2020). Tumor necrosis factor receptor family costimulation increases regulatory T-cell activation and function via NF-κB. Eur J Immunol.

[64] Vasanthakumar A (2017). The TNF receptor superfamily-NF-κB axis is critical to maintain effector regulatory T cells in lymphoid and non-lymphoid tissues. Cell Rep.

[65] Li C (2018). TCR transgenic mice reveal stepwise, multi-site acquisition of the distinctive fat-Treg phenotype. Cell.

[66] Delacher M (2020). Precursors for nonlymphoid-tissue Treg cells reside in secondary lymphoid organs and are programmed by the transcription factor BATF. Immunity.

[68] Grinberg-Bleyer Y (2010). Pathogenic T cells have a paradoxical protective effect in murine autoimmune diabetes by boosting Tregs. J Clin Invest.

[69] Nguyen DX, Ehrenstein MR (2016). Anti-TNF drives regulatory T cell expansion by paradoxically promoting membrane TNF-TNF-RII binding in rheumatoid arthritis. J Exp Med.

[70] Kleijwegt FS (2010). Critical role for TNF in the induction of human antigen-specific regulatory T cells by tolerogenic dendritic cells. J Immunol.

[71] Saxena V (2022). Treg tissue stability depends on lymphotoxin beta-receptor- and adenosine-receptor-driven lymphatic endothelial cell responses. Cell Rep.

[72] Itahashi K (2022). BATF epigenetically and transcriptionally controls the activation program of regulatory T cells in human tumors. Sci Immunol.

[73] Cretney E (2011). The transcription factors Blimp-1 and IRF4 jointly control the differentiation and function of effector regulatory T cells. Nat Immunol.

[74] Dias S (2017). Effector regulatory T cell differentiation and immune homeostasis depend on the transcription factor Myb. Immunity.

[75] Sekiya T (2022). Comparison between Nr4a transcription factor regulation and function in lymphoid and tumor Treg cells. Front Immunol.

[76] Rudra D (2012). Transcription factor Foxp3 and its protein partners form a complex regulatory network. Nat Immunol.

[77] Kidani Y (2022). CCR8-targeted specific depletion of clonally expanded Treg cells in tumor tissues evokes potent tumor immunity with long-lasting memory. Proc Natl Acad Sci U S A.

[78] Barsheshet Y (2017). CCR8^+^FOXp3^+^ T_reg_ cells as master drivers of immune regulation. Proc Natl Acad Sci U S A.

[79] Kishore M (2017). Regulatory T cell migration is dependent on glucokinase-mediated glycolysis. Immunity.

[80] Haas R (2016). Intermediates of metabolism: from bystanders to signalling molecules. Trends Biochem Sci.

[81] Torrey H (2020). A novel TNFR2 agonist antibody expands highly potent regulatory T cells. Sci Signal.

[82] Tilburgs T (2015). Human HLA-G+ extravillous trophoblasts: Immune-activating cells that interact with decidual leukocytes. Proc Natl Acad Sci U S A.

[83] Robinson MD, Oshlack A (2010). A scaling normalization method for differential expression analysis of RNA-Seq data. Genome Biol.

[84] Zhou Y (2019). Metascape provides a biologist-oriented resource for the analysis of systems-level datasets. Nat Commun.

