# Supplementary material for: Tregs from human blood differentiate into nonlymphoid tissue–resident effector cells upon TNFR2 costimulation
Source: JCI Insight. 2024 Jan 30;9(5):e172942. doi: 10.1172/jci.insight.172942 (PMC10972588; doi:10.1172/jci.insight.172942)
Supplement: Supplemental data [file jciinsight-9-172942-s149.pdf]

Supplemental Information

Treg cells from human blood differentiate into non-lymphoid tissue-resident effector cells upon TNFR2 costimulation

Mark Mensink[1], Lotte J. Verleng[1], Ellen Schrama[1],

George M.C. Janssen[2], Rayman T.N. Tjokrodirijo[2], Peter A. van Veelen[2],

Qinyue Jiang[4], M. Fernanda Pascutti[4], Marie-Louise van der Hoorn[3],

Michael Eikmans[4], Sander de Kivit[1], and Jannie Borst[1,*]

[1]Department of Immunology and Oncode Institute, Leiden University Medical Center, Leiden, The Netherlands

[2]Center for Proteomics and Metabolomics, Leiden University Medical Center, Leiden, The Netherlands

[3]Department of Obstetrics and Gynaecology, Leiden University Medical Center, Leiden, The Netherlands

[4]Department of Immunology, Leiden University Medical Center, Leiden, The Netherlands

*Correspondence: Prof. Jannie Borst, Albinusdreef 2, 2333 ZA Leiden, The Netherlands.

Phone: +31 71 5263827. Email: j.g.borst@lumc.nl

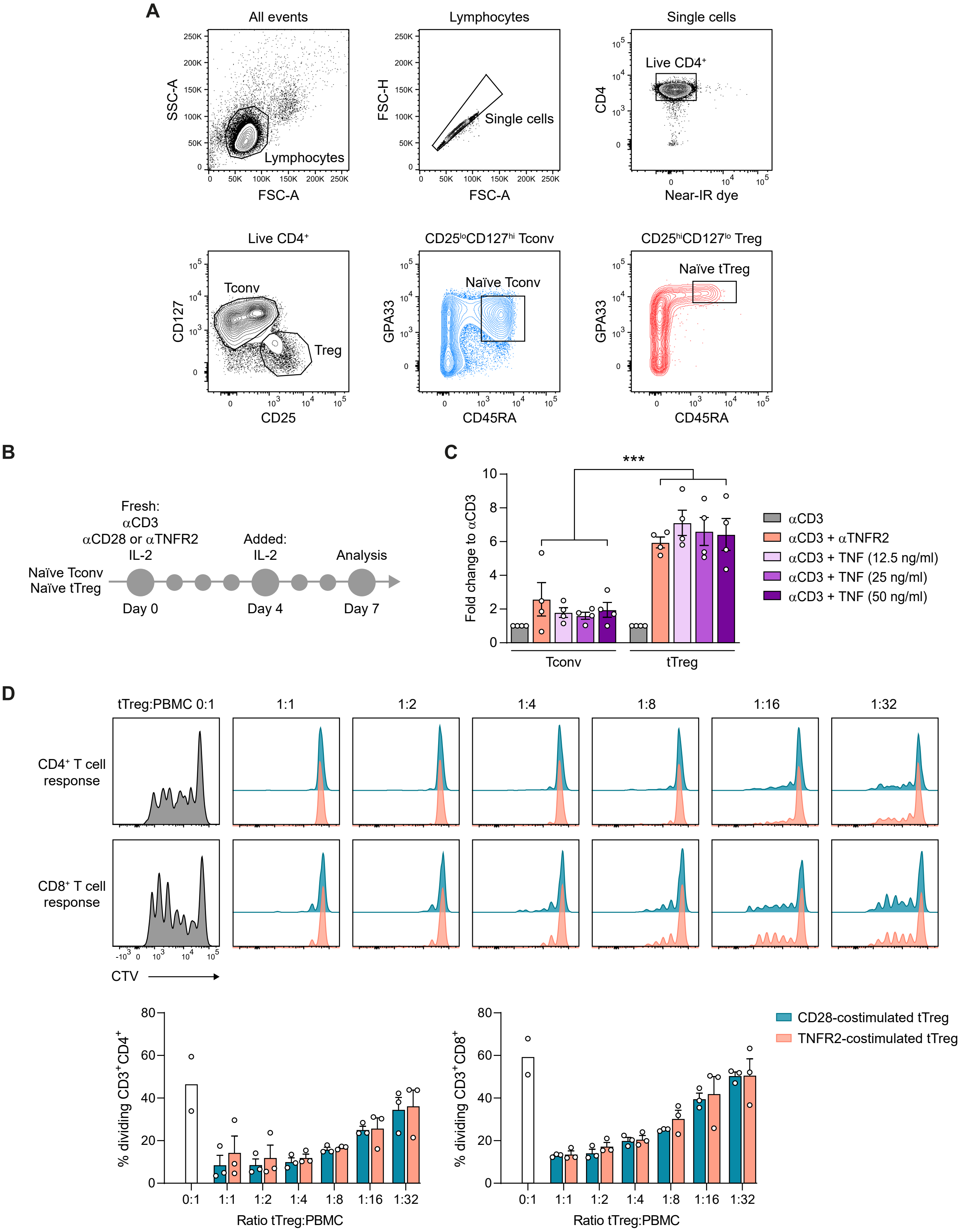

Supplemental Figure 1. Cell sorting strategy, cell culture and assessment of suppressive function.
(A) Total human CD4⁺ T cells were isolated from peripheral blood by magnetic-activated cell sorting (MACS) and subjected to FACS. Among single cell and live (near-IR dye⁻) CD4⁺ cells, total Tconv and Treg cells were distinguished based on a CD25^{lo}CD127^{hi} or CD25^{hi}CD127^{lo} phenotype, respectively. Naïve Tconv (blue) and tTreg cells (red) were sorted based on high CD45RA expression. GPA33 was used as an additional marker to obtain naïve tTreg cells to high purity (1, 2). **(B)** Schematic overview of naïve Tconv and tTreg cell cultures after cell sorting. **(C)** Tconv and tTreg cell proliferation in response to agonistic anti-CD3 mAb combined with either agonistic anti-TNFR2 mAb or recombinant TNF (12.5-50 ng/ml). Proliferation was measured at day 7 using count beads and normalized to anti-CD3 mAb alone. Statistical analysis by two-way ANOVA with Tukey's post hoc test (n=4). ***p<0.001. **(D)** Assessment of the suppressive capacity of CD28- or TNFR2-costimulated tTreg cells cocultured at different ratios with CTV-labeled PBMCs in a standard suppression assay. Cells in the suppression assay were stimulated with agonistic anti-CD3 mAb and analyzed after 4 days. The percentage of dividing CD4⁺ and CD8⁺ T cells is shown (n=3). **(C and D)** Data are presented as mean ± SEM. Sample size (n) represents individual donors, analyzed in independent experiments.

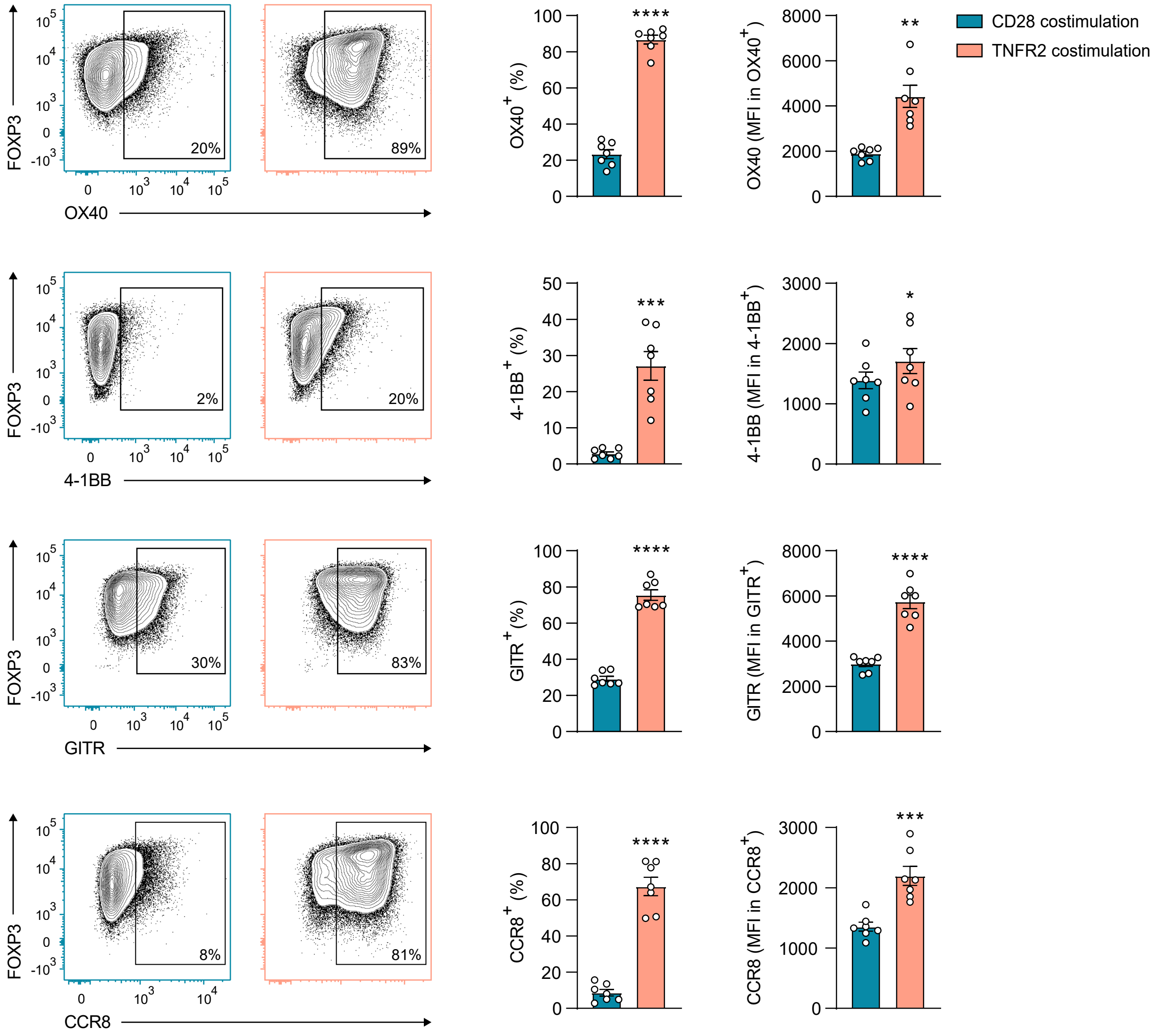

Supplemental Figure 2. Regulation of cell surface molecules in tTreg cells upon costimulation.
Naïve tTreg cells were activated with anti-CD3 mAb and costimulated with agonistic mAb to either CD28 or TNFR2 for 7 days and analyzed by flow cytometry. Representative plots and quantified protein expression are shown for the indicated molecules (*n*=7). Data are quantified as percentage of positive cells as indicated by gates. MFI within the positive fraction is also shown. Statistical analysis was done by paired two-sided Student's t-test. Data are presented as mean ± SEM. Sample size (*n*) represents individual donors, analyzed in independent experiments. **p*<0.05, ***p*<0.01, ****p*<0.001, *****p*<0.0001.

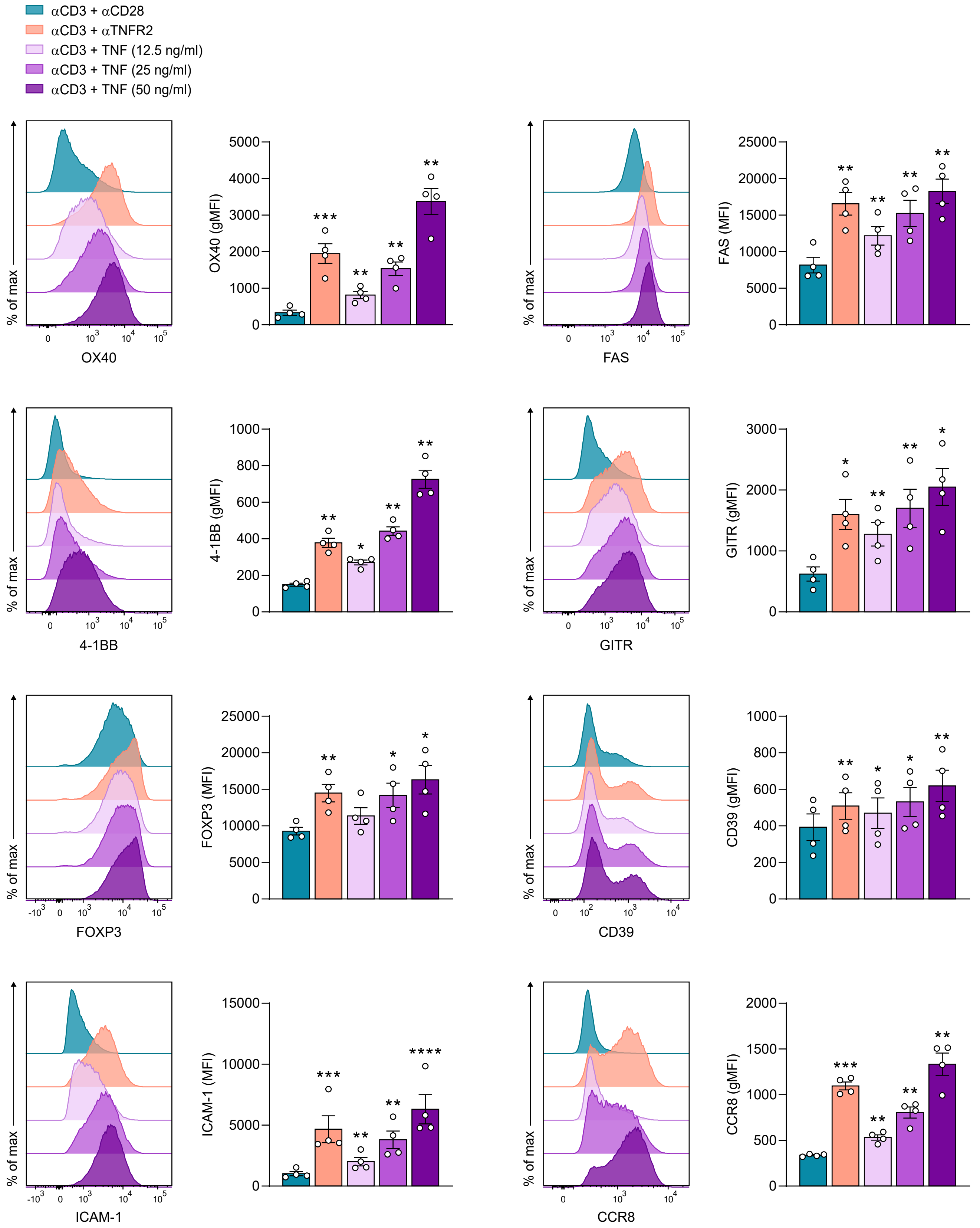

Supplemental Figure 3. TNF increases the expression of Treg cell-associated proteins in a dose-dependent manner.
Naïve tTreg cells were activated with anti-CD3 mAb combined with agonistic mAb to either CD28 or TNFR2 or recombinant human TNF (12.5-50 ng/ml) for 7 days and analyzed by flow cytometry. Representative plots and quantified protein expression are shown for the indicated molecules (*n*=4). Data are quantified as MFI or gMFI. Statistical analysis was done by one-way repeated measures ANOVA with Dunnett's post hoc test, with comparisons to CD28 costimulation. Data are presented as mean ± SEM. Sample size (*n*) represents individual donors, analyzed in independent experiments. *$p<0.05$, **$p<0.01$, ***$p<0.001$, ****$p<0.0001$.

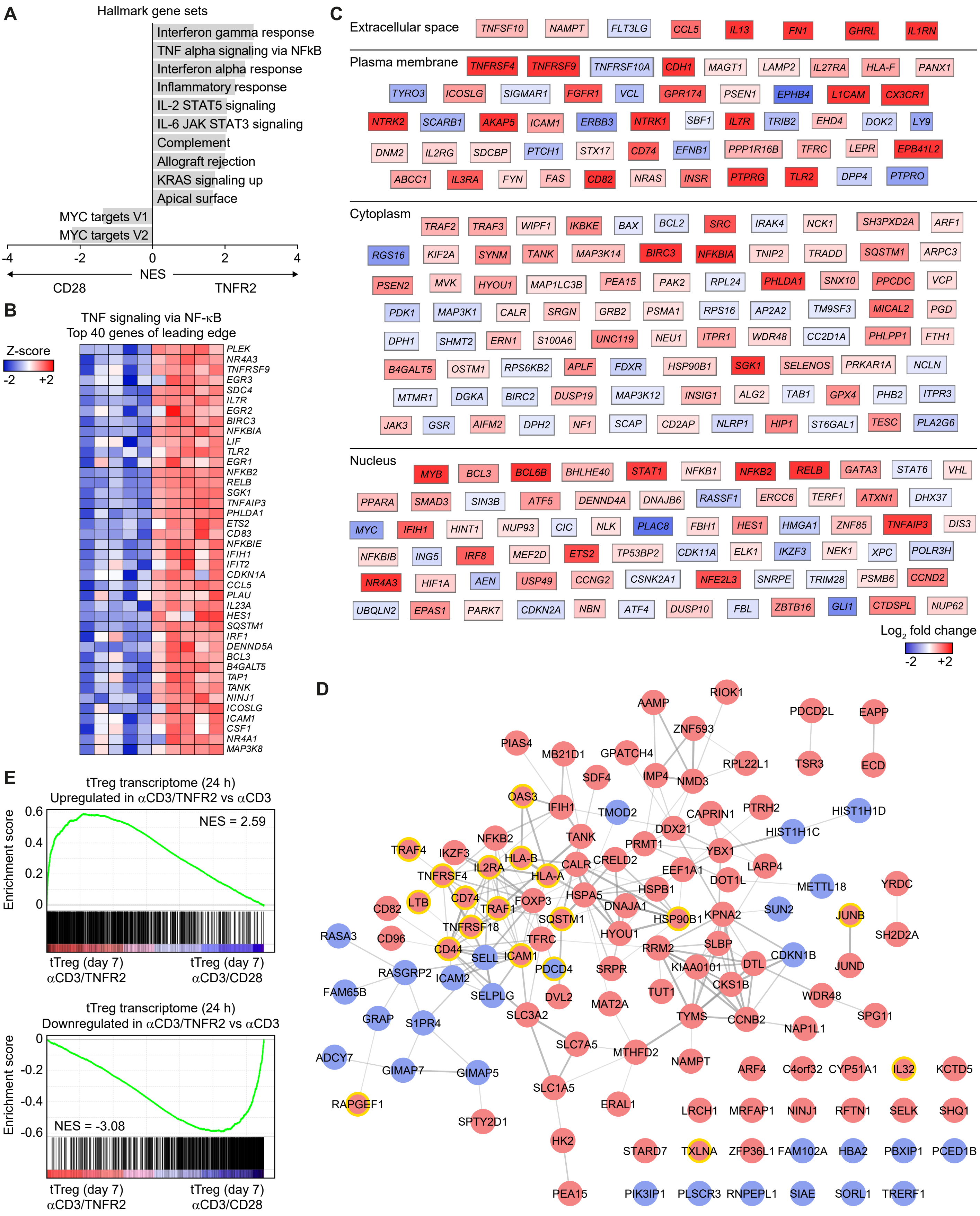

Supplemental Figure 4. Transcriptomic responses of CD28- and TNFR2-costimulated tTreg cells.
(A) GSEA using Hallmark gene sets to identify biological processes associated with the transcriptome of tTreg cells costimulated via CD28 or TNFR2 for 7 days. The top 10 gene sets significantly enriched in TNFR2-costimulated tTreg cells are shown, as well as the 2 significantly enriched gene sets in CD28-costimulated tTreg cells (FDR<0.05). **(B)** Heat map showing the top 40 genes of the leading edge from the enriched gene set "TNF signaling via NF-κB" in the transcriptome of TNFR2-costimulated tTreg cells. Z-scores are color-coded. **(C)** Differentially expressed genes in TNFR2- compared to CD28-costimulated tTreg cells associated with cell survival, according to IPA. Log_2 fold changes are color-coded. **(D)** STRING network of proteome data showing proteins differentially expressed in pre-expanded tTreg cells activated via CD3 and costimulated via TNFR2 or CD28 for 24 h by (p<0.05, 1.25-fold change). Red and blue nodes depict up- and downregulation upon TNFR2 costimulation, respectively. Lines represent associations, with thickness indicating confidence for predicting interaction. Proteins involved in cytokine-mediated signaling according to GO are labeled by a yellow outline. **(E)** GSEA using up- or downregulated genes identified in a previously transcriptomics analysis (1, 3) in pre-expanded tTreg cells that were activated via CD3 and costimulated via TNFR2 or CD28 for 24 h. Enrichment is shown in the transcriptome of tTreg cells costimulated via TNFR2 or CD28 for 7 days (FDR<0.05).

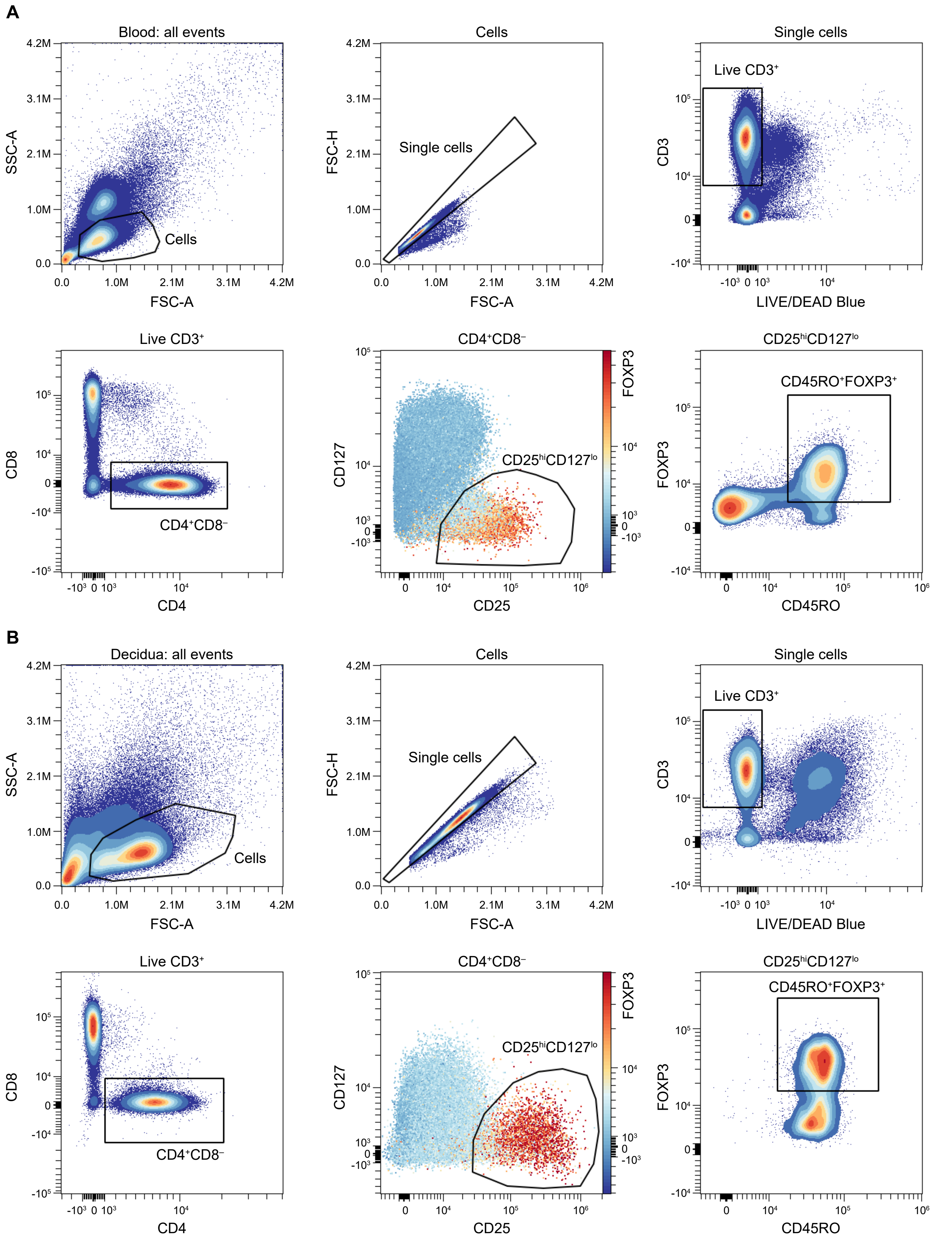

Supplemental Figure 5. Gating strategy for spectral flow cytometry analysis of human blood and decidua Treg cells.
(A and B) Representative spectral flow cytometry gating strategies to analyze effector Treg cells among lymphocytes from human maternal peripheral blood **(A)** and matching decidua parietalis **(B)**. Live $CD4^+$ T cells were selected based on single cell, live (near-IR dye$^-$) $CD3^+$ and $CD4^+CD8^-$ gates. Next, Treg cells were distinguished based on a $CD25^{hi}CD127^{lo}$ phenotype. Relative expression of FOXP3 is color-coded and shown to confirm selection of Treg cells. Finally, $CD45RO^+FOXP3^+$ effector phenotype Treg cells were selected for further analysis.

Supplemental Table 1. List of the 1229 differentially expressed genes between tTreg cells costimulated via TNFR2 or CD28 for 7 days.

Supplemental Table 2. Descriptions of published gene sets used for GSEA and summary of results (Tab 1), including selection of gene sets for the generation of combined NLT or LT Treg cell signatures (Tab 2).

Supplemental Table 3. Combined healthy and diseased NLT and LT Treg cell signatures and corresponding GSEA leading edges.

Supplemental Table 4. Clinical characteristics of women included for placenta and maternal peripheral blood analysis.

Supplemental Table 5. All antibodies and other reagents used for flow cytometry.

Supplemental Methods

Bulk transcriptomics

CD28- or TNFR2-costimulated Tconv and tTreg cells (1 × 10^5 per sample) were collected on day 7 of cell culture, washed in ice-cold PBS and resuspended in RLT buffer (Qiagen). Total RNA isolation was performed according to the manufacturer's protocol using the RNeasy MinElute Cleanup Kit (Qiagen) including an on-column DNAse digestion (Qiagen). Quality and quantity of total RNA were assessed on a 2100 Bioanalyzer using a Nano chip (Agilent). From RNA samples with a measured RNA Integrity Number (RIN) between 8.0 and 10.0, strand-specific libraries were generated using the TruSeq Stranded mRNA sample preparation kit (Illumina Inc.) according to the manufacturer's instructions (Illumina, Part #15031047 Rev. E). Polyadenylated RNA from intact total RNA was purified using oligo-dT beads. Following purification, RNA was fragmented, randomly primed and reverse-transcribed using SuperScript II Reverse Transcriptase (Invitrogen). Second strand synthesis was performed using Polymerase I and RNAse H with replacement of dTTP for dUTP. Generated cDNA fragments were 3'-end-adenylated, ligated to Illumina Paired-end sequencing adapters and amplified by 12 PCR cycles. Libraries were analyzed on a 2100 Bioanalyzer using a 7500 chip (Agilent), diluted and pooled equimolar into a multiplex sequencing pool. Pooled libraries were sequenced with 65 bp single end reads on a HiSeq2500 using V4 chemistry (Illumina Inc.). The 65 bp single end reads were mapped to the human reference genome (hg38, genome_snp_tran) using HISAT2 (version 2.1.0). Read counts were generated using Gensum (https://github.com/NKI-GCF/gensum). Ensembl GTF version 102 was used as a reference.

Mass spectrometry (MS) and proteome analysis

Naïve tTreg cells were pre-expanded for 7 days using CD28 costimulation as described in the **Methods** and were cultured for 4 additional days in absence of agonistic mAbs and in presence of IL-2. Next, 1 × 10^6 cells were cultured for 24 h in 24-well plates, in presence of IL-2, anti-CD3 mAb and either anti-CD28 or anti-TNFR2 mAb. Restimulated cells were washed in ice-cold PBS and lysis, digestion and tandem mass tag (TMT) labeling were performed as described (4). Lysis was performed using 5% SDS lysis buffer (100 mM Tris-HCl pH 7.6) and 5 U of benzonase nuclease (Thermo Scientific) by 4 min incubation at 95°C. Protein concentration was determined using Pierce BCA Gold protein assay (Thermo Scientific). 100 μg of protein of each sample was reduced with 5 mM TCEP. Reduced disulfide bonds were alkylated using 15 mM iodoacetamide. Excess iodoacetamide was quenched using 10 mM DTT. Protein lysates were precipitated using chloroform/methanol. Pellets were resolubilized in 40 mM HEPES pH 8.4 and digested using TPCK-treated trypsin (1:12.5 enzyme:protein ratio) overnight at 37°C. Peptide concentration was determined using Pierce BCA Gold protein assay.

Peptides were labeled with TMTpro Label Reagents (Thermo Scientific) in a 1:4 ratio by mass (peptides:TMT reagents), for 1 h at room temperature. Excess TMT reagent was quenched with 5 μl of 6% hydroxylamine for 15 min at room temperature. Samples were pooled and lyophilized. The TMT sample set was dissolved in 1 ml of 10 mM ammonium bicarbonate and fractionated using 1cc C18 SPE cartridges (Oasis HLB, Waters) with 15%, 17.5%, 20%, 25% and 35% acetonitrile buffers in 10 mM ammonium bicarbonate pH 8.4. TMT-labeled peptides were dissolved in water/formic acid (100/0.1 v/v) and subsequently analyzed twice by on-line C18 nanoHPLC MS/MS with a system containing an UltiMate3000 HPLC system and an Exploris480 mass spectrometer (Thermo Scientific). Fractions were injected onto a cartridge precolumn (300 μm × 5 mm,

C18 PepMap, 5 μm, 100 A) and eluted via a homemade analytical nano-HPLC column (50 cm × 75 μm, Reprosil-Pur C18-AQ 1.9 μm, 120 A (Dr Maisch, Ammerbuch, Germany)). The gradient was run from 2% to 30% solvent B (20:80:0.1 water:acetonitrile:formic acid v/v) in 240 min. The nano-HPLC column was drawn to a tip of 10 μm and acted as electrospray needle of the MS source. The mass spectrometer was operated in data-dependent MS/MS mode with 3 s cycle time, with HCD collision energy at 36 V and recording of the MS2 spectrum in the orbitrap, with a quadrupole isolation width of 1.2 Da. In the master scan (MS1), resolution was 120,000, scan range 350-1200, and standard AGC target at maximum fill time of 50 ms. A lock mass correction on the background ion m/z=445.12 was used. Precursors were dynamically excluded after *n*=1 with an exclusion duration of 45 s, and with a precursor range of 20 ppm. Charge states 2-5 were included. For MS2 the first mass was set to 110 Da, and the MS2 scan resolution was 45,000 at an AGC target of 200% at maximum fill time of 60 ms.

Raw data were converted to peak lists using Proteome Discoverer software (version 2.4, Thermo Scientific), and submitted to UniProt (Homo sapiens, 20596 entries), using Mascot version 2.2.07 (www.matrixscience.com) for protein identification. Mascot searches were performed with 10 ppm and 0.02 Da deviation for precursor and fragment mass, respectively, and the enzyme trypsin was specified. Up to two missed cleavages were allowed. Methionine oxidation and acetyl on protein N-terminus were set as fixed modifications. Protein and peptide FDR were set to 1%. Normalization was on the total peptide amount. Identification of differentially expressed proteins with at least one unique peptide was performed using Proteome Discoverer. One CD28-costimulated tTreg cell sample was excluded from the analysis due to technical reasons. Differentially expressed proteins were visualized as a network using the STRING app within Cytoscape software.

References

1. de Kivit S, et al. Stable human regulatory T cells switch to glycolysis following TNF receptor 2 costimulation. *Nat Metab.* 2020;2(10):1046-61.
2. Opstelten R, et al. GPA33: a marker to identify stable human regulatory T cells. *J Immunol.* 2020;204(12):3139-48.
3. Mensink M, et al. TNFR2 costimulation differentially impacts regulatory and conventional $CD4^+$ T-cell metabolism. *Front Immunol.* 2022;13:881166.
4. Paulo JA, Gygi SP. Nicotine-induced protein expression profiling reveals mutually altered proteins across four human cell lines. *Proteomics.* 2017;17(1-2).
