# Supplementary material for: Tregs from human blood differentiate into nonlymphoid tissue–resident effector cells upon TNFR2 costimulation
Source: JCI Insight. 2024 Jan 30;9(5):e172942. doi: 10.1172/jci.insight.172942 (PMC10972588; doi:10.1172/jci.insight.172942)
Supplement: Supplemental table 4 [file jciinsight-9-172942-s153.pdf]

**Maternal age** (years), mean (SD)	33.75 (3.77)
**Gestational age** (weeks), mean (SD)	37.8 (1.50)
**Gravity**, mean (SD)	3.5 (1.29)
**Parity**, mean (SD)	1.5 (0.57)
**Mode of delivery** (%)	Caesarean section (75%) / Spontaneous (25%)
**Sex of child** (%)	Female (100%)
**Fetal birth weight** (grams), mean (SD)	3067 (322.09)

Supplemental Table 4. Clinical characteristics of women included for placenta and maternal peripheral blood analysis.
