# Supplementary material for: Tregs from human blood differentiate into nonlymphoid tissue–resident effector cells upon TNFR2 costimulation
Source: JCI Insight. 2024 Jan 30;9(5):e172942. doi: 10.1172/jci.insight.172942 (PMC10972588; doi:10.1172/jci.insight.172942)
Supplement: Supplemental table 5 [file jciinsight-9-172942-s154.pdf]

Materials for flow cytometry	Clone	Source	Catalog number
Mouse anti-CD3	CLB-T3/4.E, 1XE, IgE isotype	Sanquin	M1654
Mouse anti-CD28	CLB-CD28/1, 15E8	Sanquin	M1650
Mouse anti-TNFR2	MR2-1	Hycult Biotech	HM2007
Mouse anti-CD3-AF700	UCHT1	BioLegend	300424
Mouse anti-CD3-BV510	UCHT1	BioLegend	300448
Mouse anti-CD3-PE	UCHT1	Dako	R0810
Mouse anti-CD3-AF532	RPA-T4	Invitrogen	58-0049-42
Mouse anti-CD4-FITC	HIT4a	ImmunoTools	21810043
Mouse anti-CD4-PE-Cy7	OKT4	BioLegend	317414
Mouse anti-CD4-SparkUV387	SK3	BioLegend	344685
Mouse anti-CD8-APC	HIT8a	ImmunoTools	21810086
Mouse anti-CD8-SparkBlue550	SK1	BioLegend	344760
Mouse anti-CD19-RY586	SJ25C1	BD Biosciences	568107
Mouse anti-CD25-BV421	BC96	BioLegend	302630
Mouse anti-CD25-PE	2A3	BD Biosciences	341011
Mouse anti-CD25-PE-Fire700	M-A251	BioLegend	356145
Mouse anti-CD26-PE	L272	BD Biosciences	340423
Mouse anti-CD39-BV510	A1	BioLegend	328220
Mouse anti-CD45-NovaFluor-Blue610-30S	2D1	Invitrogen	H005T03B05
Mouse anti-CD45RA-FITC	HI100	ImmunoTools	21819453
Mouse anti-CD45RA-PerCP	HI100	BioLegend	304156
Mouse anti-CD45RO-BV570	UCHL1	BioLegend	304226
Mouse anti-CD73-BV421	AD2	BD Biosciences	562430
Mouse anti-CD127-APC-Fire810	A019D5	BioLegend	351373
Mouse anti-CD127-BV421	A019D5	BioLegend	351310
Mouse anti-CD127-BV711	A019D5	BioLegend	351328
Mouse anti-4-1BB-BV711	4B4-1	BioLegend	309832
Mouse anti-BCL6-R718	K112-91	BD Biosciences	566979
Mouse anti-Biotin-PE	1D4-C5	BioLegend	409003
Mouse anti-CCR4-BUV615	1G1	BD Biosciences	613000
Mouse anti-CCR6-BB700	11A9	BD Biosciences	566477
Mouse anti-CCR8-BB515	433H	BD Biosciences	566891
Mouse anti-CTLA-4-BV785	BNI3	BioLegend	369624
Mouse anti-CTLA-4-PE-Dazzle594	L3D10	BioLegend	349922
Mouse anti-CXCR3-APC-Fire750	G025H7	BioLegend	353754
Mouse anti-CXCR5-BV750	J252D4	BioLegend	356942
Rat anti-Eos-PE	W16032A	BioLegend	399303
Mouse anti-FAS-BB700	DX2	BD Biosciences	566543
Rat anti-FOXP3-APC	PCH101	Invitrogen	17-4776-42
Mouse anti-FOXP3-PE-Dazzle594	206D	BioLegend	320126
Mouse anti-GARP-BUV661	7B11	BD Biosciences	750215
Rat anti-GATA3-PE-Cy5	TWAJ	Invitrogen	15-9966-42
Mouse anti-GITR-BV421	108-17	BioLegend	371208
Mouse anti-GITR-BV650	V27-580	BD Biosciences	747663
Mouse anti-GPA33		Kindly provided by Prof. D. Amsen (Sanquin)	N/A
Armenian hamster anti-Helios-PE-Cy7	22F6	BioLegend	137236
Armenian hamster anti-Helios-PerCP-eFluor710	22F6	Invitrogen	46-9883-42
Mouse anti-HLA-DR-APC-Cy7	L243	BioLegend	307618
Mouse anti-HLA-DR-BV605	G46-6	BD Biosciences	562845

Table continued on next page

Materials for flow cytometry	Clone	Source	Catalog number
Mouse anti-ICAM-1-BB515	HA58	BD Biosciences	564685
Mouse anti-ICAM-1-BUV563	HA58	BD Biosciences	741374
Mouse anti-IFN-γ-BV750	B27	BD Biosciences	566357
Rat anti-IL-4-BV421	MP4-25D2	BioLegend	500825
Rat anti-IL-13-BV421	JES10-5A2	BioLegend	501915
Mouse anti-IL-17A-PE-Dazzle594	BL168	BioLegend	512335
Mouse anti-IL-21-AF647	3A3-N2.1	BD Biosciences	560493
Mouse anti-Ki-67-BV480	B56	BD Biosciences	566172
Mouse anti-LAYN (biotinylated)	3F7D7E2	Sino Biological	10208-MM02-B
Mouse anti-OX40-BUV737	ACT35	BD Biosciences	749286
Mouse anti-OX40-PE-Cy7	ACT35	BD Biosciences	563663
Mouse anti-PD-L2-BV421	24F.10C12	BioLegend	329615
Mouse anti-RORγt-PE	Q21-559	BD Biosciences	563081
Mouse anti-T-bet-PE-Cy7	eBio4B10	Invitrogen	25-5825-80
Mouse anti-TIGIT-BV605	A15153G	BioLegend	372712
Mouse anti-TNF-BUV395	MAb11	BD Biosciences	563996
Rat anti-TNFR2-PE-Cy7	3G7A02	BioLegend	358412
AlexaFluor647 succinimidyl ester	N/A	Invitrogen	A37573
CellTrace Violet (CTV)	N/A	Invitrogen	C34557
FOXP3 transcription factor staining buffer set	N/A	Invitrogen	00-5523-00
Human Fc-Block	N/A	BD Biosciences	564220
LIVE/DEAD™ fixable blue dead cell stain kit	N/A	Invitrogen	L34961
LIVE/DEAD™ fixable near-IR dead cell stain kit, for 633 or 635 nm excitation	N/A	Invitrogen	L10119
UltraComp eBeads compensation beads	N/A	Invitrogen	01-2222-42

Supplemental Table 5. All antibodies and other reagents used for flow cytometry.
